# Interprofessional Error Disclosure Training for Medical, Nursing, Pharmacy, Dental, and Physician Assistant Students

**DOI:** 10.15766/mep_2374-8265.10606

**Published:** 2017-07-21

**Authors:** Karen A. McDonough, Andrew A. White, Peggy Soule Odegard, Sarah E. Shannon

**Affiliations:** 1 Associate Professor, Department of Medicine, University of Washington School of Medicine; 2 Lynn and Geraldine Brady Endowed Professor of Pharmacy, University of Washington School of Pharmacy; 3 Associate Dean, University of Washington School of Pharmacy; 4 Senior Associate Dean for Academic Affairs, Oregon Health & Sciences University; 5 Professor of Nursing, Oregon Health & Sciences University

**Keywords:** Interprofessional, Standardized Patient, Error Disclosure, Interprofessional Relations, Communication Skills Training

## Abstract

**Introduction:**

Errors that harm patients often have many contributing factors and ideally should be disclosed by a team rather than an individual provider. However, most health professions students learn about errors and error disclosure in a single-profession class.

**Methods:**

We developed a 2-hour small-group session in which our students practice discussing and disclosing a medical error that involves several professions, following a communication map. As they practice, students gain an understanding of the roles, skills, and perspectives of the other professions represented in the group.

**Results:**

Over the last 5 years, student evaluations have been very positive. In 2016, our students strongly agreed that “The small group skills practice was a useful and interesting learning opportunity,” “Learning with other professional students was valuable,” and “Thinking about error disclosure from a team perspective was helpful.” Student comments consistently indicated that they learned both about disclosing medical errors as well as other professionals' roles and perspectives.

**Discussion:**

This activity has met both of our major goals. The first was to bring health professions students together to learn with, from, and about each other. The second was to practice a critical and challenging communication skill. This activity has been successfully implemented at other institutions, and can be adapted to fit other groups of students.

## Educational Objectives

By the end of this session, learners will be able to:

1. Discuss a medical error as an interprofessional team in a blame-free way.
2. Plan for a disclosure of a medical error as an interprofessional team.
3. Disclose a medical error to a simulated patient as an interprofessional team with honesty, compassion, and respect for team members.
4. Articulate each team member's role in the patient's care and each team member's contribution to a medical error.

## Introduction

Patients expect health care providers to promptly disclose mistakes and apologize for harmful errors.^1^ However, health care teams rarely meet patient expectations for communication after medical injury.^2^ For example, only a minority of errors are disclosed to patients, disclosure conversations often lack key details about what happened, and patients rarely receive the emotional support they desire.^3^ Although most medical and nursing students witness or participate in an error during their clinical training, only a minority receive training in how to approach disclosure.^4,5^ Academic health centers can enhance transparency in health care by preparing students in all professional schools for the challenge of disclosing errors.

Prior to 2011, medical students, nursing students, and pharmacy students at our institution all learned about medical errors and error disclosure in their own single profession classes with no opportunity for skills practice or exploration of the role and perspective of other team members. Each profession learned about errors independently despite the evidence that most health care is provided by interdisciplinary teams and that the majority of medical errors involve a communication failure.^6^ With support from a grant from the Josiah Macy, Jr. Foundation, we developed this session to fill an unmet need for joint learning about error disclosure. We targeted learners in the preclinical phase of their training both because it simplified the logistics of assembling the groups at a single site, and because developing these communication skills requires early exposure in a consequence-free environment. The prerequisite knowledge included basic familiarity with: (1) the learner's profession's role in the process of ordering, verifying, and administering an antibiotic, (2) the scope and nature of allergic drug reactions, and (3) the process by which patient allergies might be identified to providers in a hospital or nursing facility.

This session serves two purposes. Students practice and receive feedback on a team-based approach to disclosing a medical error. More importantly, students learn about the roles, skills, and training of each of the professionals in the group and practice open and nonhierarchical communication. Many interprofessional education activities have been shown to improve students' understanding of the roles of other professions and their team communication.^7–9^ We have found error disclosure to be a particularly powerful opportunity for practicing the core competencies for interprofessional collaborative care.

Our unique contribution provides an efficient and effective structure for practicing error disclosure in an interprofessional small group using low-fidelity simulation. Simulation is particularly conducive to teaching communication skills such as error disclosure, as these types of exchanges carry major emotional and legal implications. Practice in a consequence-free environment is therefore essential for novice learners.^10^ Prior authors have developed OSCEs or standardized patient cases in which residents practice or are assessed in error disclosure skills.^11,12^ This activity allows a larger group of interprofessional students to practice this critical skill with a single patient actor, and to receive feedback from a facilitator and peers in addition to the standardized patient.

## Methods

This is a 2-hour small-group session for approximately 12 interprofessional students. At a minimum, this case requires medical, nursing, and pharmacy students to make the case work as written. The case includes optional roles for dental and physician assistant students and can be adapted to some degree to fit the needs of other learners. Didactic content is delivered prior to the session to preserve in-class time for skills practice. Students are expected to watch a 20-minute video (Appendix A) and read the recommended preparatory article.^1^

The session follows the itinerary in the Facilitator Guide (Appendix B). After introductions and an icebreaker exercise, students receive profession-specific information (Appendix C) about the case of Albert, an elderly man admitted from a nursing facility with pneumonia. Albert had an unrecognized penicillin allergy, and suffered anaphylaxis after being treated with piperacillin/tazobactam. To assist with discussion and skills practice, the students also receive a pocket card referencing the key elements of interprofessional planning and error disclosure described in the preclass video (Appendix D). After discussing the error and how it occurred, a team of three to four students disclose the error to a simulated family member. After reflecting on the disclosure, another team discloses the same error, this time receiving a different emotional response from the simulated family member. This is repeated until all groups have had a chance to practice. Finally, students reflect on the skill of error disclosure, as well as their new understanding of other professions, and team communication.

Each small group is facilitated by at least two instructors. To model interprofessional collaboration, ideally they should be from different professions. The first faculty member facilitates the session and should understand the basics of both error disclosure and interprofessional facilitation. The second faculty member plays the role of a family member to whom a medical error is disclosed. After the students practice disclosure, this faculty member also debriefs with them, offering them feedback on their teamwork, communication, and emotion handling. This role has been filled by a standardized patient or professional staff at other schools. Other than a room with adequate seating, printouts of the student materials, and a whiteboard to facilitate discussion, no equipment is required.

We prepare new faculty by distributing our facilitator guide (Appendix B) 1 week in advance. We then conduct a 1-hour just-in-time training session immediately prior to the class, intended to prepare them to run the small group and act as the family member. Total faculty time commitment needed for the session includes review of faculty manual (approximately 30 minutes on own time), just-in-time training (1 hour), and teaching session (2 hours) for a total of just under 4 hours. In subsequent academic cycles, the time requirements are approximately 1 hour less for returning faculty. Faculty have found this a reasonable time commitment that is efficiently organized to be done in one condensed time period.

This session has been taught annually since 2011, with some iterative refinements. Attendance is required for all students at the intended level of training, leading to a class size of over 500 learners. Originally, the skills practice small group was preceded by a 1 hour lecture on medical error and disclosure. Because of both space limitations and student feedback, we replaced the large lecture with the web-based video. From 2011 to 2014, small groups were made up of students from medicine, nursing, and pharmacy. In 2014, we added both dental students and physician assistant students to all groups, and dietetics students and Master of Health Administration students to some groups as observers or coaches.

Files included in this publication are:

• Preclass module (Appendix A): This is the online module that our students are assigned before class. It covers the rationale for transparency about medical errors, the role of the interprofessional team, and apology laws. Students are urged to read the recommended article prior to the session.^1^
• Error Disclosure Facilitator Guide (Appendix B): This is a detailed, step-by-step guide that describes timing, facilitation strategies, key teaching points, and potential pitfalls. It has been used to successfully implement the same session at other institutions.
• Profession-Specific Cases (Appendix C): These are distributed to students at the beginning of the session. They tell the story of the error from that profession's perspective. Students from different professions come together to discuss the case, together building a complete picture of the error and how it happened.
• Error Disclosure Pocket Cards (Appendix D): This is a communication map that is referenced in the preclass video and distributed to students at the beginning of the session. It guides their discussion and disclosure.
• Error Disclosure Slides (Appendix E): These are the slides used to create the video. They can be altered to reflect laws in your state, and either re-recorded or presented live. Participating faculty also found it useful as a refresher on content.

## Results

Over 3,000 health professions students have participated in this activity since 2011, including 1,320 medical students, 864 nursing students, 540 pharmacy students, 306 physician assistant students, 210 dental students, 48 dietetics students, and 18 Master of Health Administration students. Their evaluations show that they value the opportunity to work with students from other professions, recognize the benefit of approaching error disclosure from a team perspective, and appreciate the feedback and teaching from the facilitators. Student evaluation data are provided for the 2015 and 2016 sessions in the accompanying Table. Student comments in response to the question “What one thing will you take into your practice” emphasize two areas; how to disclose errors, and how to work within an interprofessional team.

**Table.** Student Evaluations

Item	Rating	
	2015^b^	2016^c^
The small group skills practice was a useful and interesting learning opportunity.	4.57	4.57
Learning with other professional students was valuable.	4.67	4.56
Thinking about error disclosure from a team perspective was helpful.	4.67	4.66
The facilitator feedback was helpful.	4.67	4.62
I felt I had the opportunity to participate in the small group.	4.65	4.68
Overall, the facilitators contributed to my learning.	4.70	4.65

a Five-point Likert scale (1 = *Strongly Disagree*, 5 = *Strongly Agree*).

b *N* = 559.

c *N* = 467.

Facilitators have come from the same professions as students, as well as social work and health law. Faculty from at least two professions join each group. In most cases, one faculty member is the actor, although the actor role can also be filled by administrative staff or standardized patients. Faculty supported by a Macy Foundation grant have successfully implemented or adapted the session at their home institutions, including Indiana University, the Medical University of South Carolina, and the University of North Dakota. We believe that this would be a useful addition to an interprofessional curriculum at other schools.

## Discussion

This activity has met both of our major goals, to bring health professions students together to learn with, from and about each other, and to practice the critical and challenging communication skills of error disclosure. We have been successful because core faculty from each professional program have participated in its design, faculty recruitment, implementation, and refinement. This has allowed us to maximize the relevance to each group of learners and the integration with each school's curriculum.

All programs except nursing have integrated this session into their core curriculum, as part of the clinical skills or foundations of practice course during the quarter it is offered. Students in all programs are required to attend, and attendance is recorded. We believe this is necessary to assure representation of all involved professions in all groups. Uneven attendance from participating professions is a major potential pitfall. We recommend consistent requirements for attendance across participating schools, ideally as part of a course rather than as an extra session.

We strive to match the experience levels and average confidence levels of students from different health sciences schools. In 2011, second-year physician assistant students, who had substantially more clinical experience than any other group, participated in the error disclosure session. In many groups, they took charge of the simulated disclosure and the less experienced students sat back. In 2012 and 2013, the physician assistant students did not participate, but in 2014, the first-year students joined. Their level of clinical experience was much more closely matched to the others, and discussion and participation in small groups remained fairly balanced. Senior nursing students have more clinical experience than other learners but may lack confidence in their knowledge and skills. When paired with earlier learners from other health sciences schools they have been able to demonstrate their educational preparation and develop confidence and team skills.

Some faculty may need to update their knowledge about medical errors, current laws, and recommendations for disclosure in order to facilitate this session effectively. This has been an area of rapid change in clinical practice, organizational policy, and law over the past 10–15 years. Those with outdated beliefs may inadvertently interfere with learning by suggesting the disclosure is not appropriate, legal, or wise. Offering faculty resources to address their own knowledge gaps was especially important in our first year, and we continue to provide them with the same preparatory material that students receive. We also include learning pearls specific to error disclosure in the faculty guide. We have found that with each year, faculty have become more knowledgeable in this important area in health care.

Finally, we found that even facilitators skilled in working with uniprofessional groups were unprepared to address issues common in interprofessional small groups. We added detailed tips for managing these issues to our facilitators' guide, and added an optional 3-hour interprofessional faculty development session.

Although our error disclosure session is highly regarded by both students and faculty, it is resource intensive. We use two faculty members, one to facilitate and one to act as the family member, for each group of 12 students. The session also requires a very large number of rooms.

In an attempt to build on this experience while minimizing faculty needs, we added a series of large group sessions in 2013–2014. For these additional sessions, students were seated in lecture halls in small, interprofessional teams, and worked through cases together. Student evaluations from that year demonstrated a strong preference for small-group learning. In 2014–15, we transitioned to a series of four 2-hour interprofessional small groups, beginning with the previously successful error disclosure session. Student evaluations were very positive for all small groups over the course of the academic year. We anticipate continuing this interprofessional series of small groups for early learners over the next several years.

## Appendices

A. Interprofessional Error Disclosure Module folder
B. Error Disclosure Faculty Facilitators Guide.docx
C. Profession-Specific Cases.docx
D. Error Disclosure Pocket Cards.pdf
E. Error Disclosure Slides.pptx All appendices are peer reviewed as integral parts of the Original Publication.
