# Supplementary material for: Interprofessional Error Disclosure Training for Medical, Nursing, Pharmacy, Dental, and Physician Assistant Students
Source: MedEdPORTAL. 2017 Jul 21;13:10606. doi: 10.15766/mep_2374-8265.10606 (PMC6338166; doi:10.15766/mep_2374-8265.10606)
Supplement: Supplementary file 1 — A. Interprofessional Error Disclosure Module folder B. Error Disclosure Faculty Facilitators Guide.docx C. Profession-Specific Cases.docx D. Error Disclosure Pocket Cards.pdf E. Error Disclosure Slides.pptx [file mep-13-10606-s001.zip › A. Interprofessional Error Disclosure Module folder/story.html]

Interprofessional Error Disclosure MedEd Portal
