# Supplementary material for: Interprofessional Error Disclosure Training for Medical, Nursing, Pharmacy, Dental, and Physician Assistant Students
Source: MedEdPORTAL. 2017 Jul 21;13:10606. doi: 10.15766/mep_2374-8265.10606 (PMC6338166; doi:10.15766/mep_2374-8265.10606)
Supplement: Supplementary file 1 — A. Interprofessional Error Disclosure Module folder B. Error Disclosure Faculty Facilitators Guide.docx C. Profession-Specific Cases.docx D. Error Disclosure Pocket Cards.pdf E. Error Disclosure Slides.pptx [file mep-13-10606-s001.zip › B. Error Disclosure Faculty Facilitators Guide.docx]

University of Washington

Interprofessional Error Disclosure Workshop

Karen A. McDonough MD

Associate Professor

Department of Medicine

University of Washington School of Medicine

Andrew A. White MD

Associate Professor

Department of Medicine

University of Washington School of Medicine

Peggy Odegard, PharmD, CDE

Herb and Shirley Bridge Endowed Professor of Pharmacy

Associate Dean, School of Pharmacy

University of Washington School of Pharmacy

Sarah E. Shannon, PhD, RN

Associate Professor

Department of Biobehavioral Nursing & Health Systems

University of Washington School of Nursing

**Acknowledgments:**

The authors gratefully acknowledge funding from the Josiah Macy Foundation Board Grant (B08-05: Drs. Brenda Zierler and Brian Ross, PIs) for initial development of all teaching materials, pilot testing of the learning event, and initial roll-out. In addition, the faculty development workshop was supported in part by funds from the Health Resources and Services Administration (HRSA)’s Advanced Education Nursing Program Grant Initiative (D09HP25029: PI Dr. Brenda Zierler).

The authors acknowledge the dedicated support of Debra Liner and Julie Calcavecchia.

The authors also thank the many UW schools of medicine, nursing, pharmacy, dentistry, social work and public health and UW Medicine clinical staff who contribute to teaching the next generation the challenging and critical skill of acknowledging and disclosing their errors. We are grateful to them for both their time and dedication.

The authors acknowledge the ongoing support of the University of Washington Board of Health Sciences Deans for this interprofessional learning experience for University of Washington health sciences students.

Finally, the authors offer their appreciation to the interprofessional learners who have embraced learning skills for error disclosure and thereby make transparency in the health care relationship a reality.

Page

**Agenda** ……………………………………………………………………….………………………………...... 3

**Overview** ……………………….…………………………………………………………………………………………….…... 4

**Facilitator Instructions:**

1. Set up learning environment ………………………………………………….………………….. 5
2. Describe session and distribute case …………………….…………….…………………..…… 5
3. Discuss error in large group and plan disclosure ……………..………….………..……. 6

- Learning goals
- Ideas for facilitation

1. Break into teams and plan roles …………………………………………….………….………….. 7
2. Team disclosure of error + debrief …………….……………………………….…….………… 8

- Learning goals
- Ideas for facilitation

1. Group debrief ………………………………………………………………………….……….………..…. 9

**Actor instructions:**

1. Overview of actor’s role ………………………………………………………….................................... 10
2. Case description from perspective of family member ……………..…………………..…….…..... 11
3. Emotion & Content Triggers – Family Member …………………………….……………..….………... 13
4. Family Member: Quick Summary of Triggers ……………………………………….……...….…….….. 15

**Resources:**

- Error Disclosure: Potential Learning Pearls ……………………………………………………………..…. 16
- Interprofessional Teams: Potential Learning Pearls …………………………………………………... 18
- Facilitating Interprofessional Groups: Potential Teaching Pearls ……………………..…….……19

**AGENDA**

| **OPTIONAL TRAINING SESSIONS FOR FACULTY** | | |
| --- | --- | --- |
| **Time** | **What** | **Where** |
| 1:00–2:00pm | Faculty training | **T553** |
| 2:00-2:30 pm | Meet your teaching partner | **T553** |

| **SMALL GROUPS** | | |
| --- | --- | --- |
| **Time** | **Activity** | **Supporting Materials** |
| 3:30-3:45 | Nametags and introduce yourself | Nametags in packet |
|  | Describe learning activity to group | Instructions in Faculty guide |
|  | Learners read case | Case in packet – **by professional group** |
| 3:45-3:55  (10 min) | Learners discuss error and plan for disclosure as a large group | Facilitator Faculty assists group  [Actor is outside room] |
| 3:55-4:00  (5 min) | Divide into 4 teams: 3 teams of MD’s+RN+pharm+PA; 1 team of DDS’s  Short discussion within each team to plan disclosure roles | Balance student mix as much as possible |
| 4:00–4:15  (15 min) | **First** team discloses to “family member”  5 minute debrief with first team | *“Error Disclosure: Learning Pearls”* in faculty guide |
| 4:15–4:30  (15 min) | **Second** team discloses to “family member”  5 minute debrief with second team | *“Error Disclosure: Learning Pearls”* in faculty guide |
| 4:30–4:45  (15 min) | **Third** team discloses to “family member”  5 minute debrief with third team | *“Error Disclosure: Learning Pearls”* in faculty guide |
| 4:45-5:00  (15 min) | **Dental team discloses dental aspect to family member;** 5 min debrief with DDS | *“Error Disclosure: Learning Pearls”* in faculty guide |
| 5:00-5:20  (20 min) | Debrief as large group about team practice and communication | *“Interprofessional Team Practice and Communication: Learning Pearls”* in faculty guide |
|  | Student Evaluations ! | In Faculty packet |

**OVERVIEW**

**LEARNING OBJECTIVES:**

1. Discuss a medical error in a blame-free way as an interprofessional team.
2. Plan for disclosure of a medical error as an interprofessional team.
3. Disclose a medical error as an interprofessional team with honesty, compassion and respect for team members.
4. Articulate each team member’s role in this patient’s care and each team member’s contribution to a medical error.

**WHAT:** Groups of two faculty and ~ 12-14 interprofessional students will practice error disclosure. The case is a missed penicillin allergy in a patient transferred from a nursing facility who receives pipercillin/tazobactam (Zosyn) in ED. The error involves all members of the ED health care team. The patient was seen earlier in day for routine dental appointment where emerging pneumonia was missed. The students will break into **FOUR** teams. Each team will talk with the patient’s ‘family member’. Prior to the session, students will have watched a 15 minute video about interprofessional error disclosure and apology.

**FACULTY ROLES (2):**

- Facilitator Role: This person is familiar with how to facilitate skills practice and interprofessional learning. He/she will facilitate the learners’ communication with each other, with the “family member” and guide the final debrief.
- Actor Role: This person will play the role of a family member of a patient who has been harmed by a medical error, to whom teams will disclose the error. This person’s key role is to be a realistic family member for the learners to practice communication skills. At the end of the session, this faculty member will provide feedback in the debrief. This faculty member should stay outside the room until he/she is brought in for disclosure and at the end for the debrief.

**COMPOSITION OF STUDENT GROUPS**:

- 4-6 2^nd^ year medical students
- 2-3 senior BSN/ABSN students
- 2-3 3^rd^ year pharmacy students
- 1 1^st^ year PA student
- 2 3^rd^ year dental students

**MATERIALS FOR STUDENTS:**

1. Nametags: Ask students to write name AND PROFESSION (ie, MD, RN, PA, Pharm, DDS).
2. Pocket Guide: Quick reference for basics of team error disclosure
3. Case: Six versions – physician, nurse, PA, pharmacist, hospital administrator and dentist.

**Please distribute the correct version to learners – they differ slightly.**

1. Evaluation: One-page evaluation with return envelope. Please collect evaluations in envelope and return via campus mail. Thank you!
2. Prereading: Gallagher, TH. A 62-Year-Old Woman With Skin Cancer Who Experienced Wrong-Site Surgery. Review of Medical Error. JAMA. 2009;302(6):669-677FACILITATOR INSTRUCTIONS:
3. **SET UP LEARNING ENVIRONMENT (10 minutes):**

**GOAL:**

- **Learners will interact with students from other professions to gain some understanding of their training**

1. **Room set up**: Group 4-5 chairs at one end of your room. This will be your ‘stage’. As each team practices, have them physically move to these chairs to interact with the family member. Ideally, this area should be near the door to allow the family member to enter and exit easily. Be sure you can see a clock from where you sit to ensure each group gets practice time.
2. **Introductions**:

- Facilitator: Share your name, school you are from, and your clinical specialty.
- Students: Ask students to introduce themselves by sharing name, their profession, and one “interesting fact” about their profession’s training.

1. **DESCRIBE LEARNING ACTIVITY (5 min):**
2. **Describe learning activity**:

- *“We are going to practice communication skills for disclosing an error to a patient or family member.*
- *“I’m going to give you a case that involved everyone here. We will take a couple minutes together to discuss the case details and talk about how this error happened and what to do next.*
  - *“Then, we’ll break into four small teams and you will have a few minutes in your teams to plan how your small team will disclose this error.*
- *“When you are ready to disclose the error, another faculty member will come in to be the patient’s family member.*
- *“Each small team will have 10 minutes to disclose the error and debrief a bit.*
- *“After each team has had a turn, we’ll spend about 15 minutes talking together about error disclosure.“*

1. **Distribute materials and allow learners time to read**:

- Case (different versions for each professional group)
- Pocket Guide

1. **DISCUSS ERROR & PLAN DISCLOSURE IN LARGE GROUP (7 min)**

**GOALS:**

- **Relevant information and concerns about the error/s are raised in the group discussion through open, non-blaming conversation.**
- **Team thinks through the actual disclosure to anticipate handling patient emotion, patient blame, etc.**

**Key Steps in Team Error Disclosure:**

|  |  |
| --- | --- |
| ***Team Discusses the Error*** | 1. Acknowledges error |
|  | 2. Conducts blame-free communication during team conversation |
|  | 3. Demonstrates team-oriented communication |
|  | 4. Negotiates differences of opinion collaboratively |
|  | 5. Recognize emotional impact of errors on team members |
|  |  |
| ***Team***  ***Plans the Disclosure*** | 1. Advocates for full disclosure |
|  | 2. Plans roles for disclosure |
|  | 3. Anticipate patient’s questions and reactions |
|  | 4. Plans responses to patient |

**IDEAS FOR FACILIATION:**

**Encourage acknowledgment of error:** be curious and non-judgmental.

*“How are allergy alerts recorded in the computer system?”*

*“In case the patient/family member asks, it might be helpful to understand the process of how medications are given. Could someone walk me through it step by step?”*

*“How would nursing usually double-check an allergy?”*

*“What type of physical assessment would a dentist normally do before treating an elderly patient?”*

**Avoid blame and help learners take personal accountability:** model blame-free communication.

Student says: “As a nurse, *I should have checked the medical record more carefully. If I had, I would have seen the drug allergy.”* Reply: *“What other checks and cross-checks in this patient’s care might have caught this allergy.”*

Student says: *“I think pharmacy is supposed to record these allergies so that the information is available when the doc is trying to order meds.”* Reply: *“That is one check but what are others?”*

Student says: *“I guess I am surprised that a health professional, even a dentist, would miss the signs of pneumonia.”* Reply: *“We all feel bad when we miss symptoms or diagnoses. As dental students, could you share some of the training you get in assessment of elderly patients such as this patient?”*

**Address emotion in colleagues:** be respectful and supportive.

*Student says: “I think I would feel awful about this.”* Reply: *“I bet you aren’t alone in that feeling. Are others feeling responsible for this error?”*

**Explore differences among all team members:** be respectful and encouraging.

*“I’m wondering if everyone has had the chance to express their perspective. It is helpful to hear your thoughts, particularly before we talk with the patient/family.”*

**Advocate for full disclosure:** support honesty

Student says: “*I’m not sure we should tell the patient/family member everything. This wasn’t entirely our fault. The SNF played a role.”* Reply: *“These situations are often complex. What do others think? What would you want to be told if this had happened to your parent?”*

**Anticipate family member’s questions and reactions and plan responses:**

*“How do you think the patient/family member may react when you tell them about this? What if he/she gets really upset or cries?” What if he/she gets angry?”*

*“What should we do if the patient/family member blames one of us?”*

*“What if they have questions we cannot answer today?”*

**IV. PLAN ROLES FOR DISCLOSURE (8 minutes)**

1. **Break group into four teams**:

- *“Let’s break into our four teams – three teams will be from the acute care setting and one will be from the dental setting.*
- *“Medical, nursing, PA, pharmacy, and dietetics students – let’s divide you into three balanced interprofessional teams”.*
- *Dental students – you will be our fourth team.”*

1. **Ask teams to discuss their plans for the disclosure. Remind them to consider roles:**

- *“Spend a few minutes talking in your teams about how you would like to handle this disclosure.*
- *“Think about who will begin the conversation. Is there specific information each of you would like to share with the family member?”*

**V. TEAMS DISCLOSE ERROR (10 min per team followed by 5 min debrief ) GOAL:**

- **Teams disclose the error honestly, clearly and compassionately demonstrating respect for all team members (present or not present).**

**Key Step in Team Error Disclosure:**

|  |  |
| --- | --- |
| ***Team Discloses Error to Patient*** | 1. Conducts explicit disclosure of error to patient |
|  | 2. Responds forthrightly to patient questions about event |
|  | 3. Apologizes upfront and early in conversation |
|  | 4. Conducts blame-free disclosure, acknowledges personal role |
|  | 5. Offers plans to prevent future errors |
|  | 6. Plans follow up with patient |

**PROCESS:**

1. Each team has 10 minutes for their disclosure to the family member. The dental team will go last.
2. Ask for a team to volunteer to be first. Thank them!
3. Move them, if possible, to a circle of chairs with place for the family member.
4. Bring actor into the room, indicate his/her chair, and introduce to the team. Be formal in referring to the actor as the family member.
5. Debrief after each team practices:

- The rest of the group will listen to this debrief, not comment. They will have their chance to debrief their own skills practice.
- Remember to allow each team member who just practiced to make a comment, not just the ‘most talkative’ person or most dominant.
- The actor can either stay in the room (head down and not interacting) or can leave the room and re-enter for the next team. (Leaving the room is best.)

DEBRIEF QUESTIONS:

1. *What went well (what worked well) when you talked with the patient’s family?*
2. *What could have gone better?*
3. *What is one thing you’d like to be sure to do the next time you disclose an error?*
4. **Orientation for change in setting for fourth (dental) team:**

- *“Albert has been discharged from the hospital and the discharge summary was sent to Albert’s dentist at his family’s request. The family member requested a meeting with the dentist/s whom Albert has seen for several years. The family member wants to understand what happened the day Albert was seen.”*

**VI. DEBRIEF AS LARGE GROUP (20 min)**

**GOALS:**

- **Consider strengths and challenges of open disclosure of medical errors.**
- **Consider strengths and challenges of interprofessional teams around error disclosure.**

1. **Start with the actor still in role as the family member:**

*“How did it feel hearing about the error in Albert’s care from each of these teams?*

1. **Bring the actor out of “role”:**

After the ‘family member’ has provided affirmative feedback, ‘introduce’ them by their actual name and profession, thank them, and elicit additional perspectives from them.

1. **Solicit reflections and feedback from students, summarizing and reflecting their comments to make key points:**

See “Error Disclosure: Learning Pearls” and “Interprofessional Teams: Learning Pearls” at the end of this manual. In this portion of the debrief, bring in the interprofessional learning pearls in addition to the error disclosure learning pearls.

1. **Evaluations:**

Please distribute evaluations to learners and have them return to the self-addressed envelope. Please place envelope in campus mail.

Thank you!

ACTOR INSTRUCTIONS:

1. **OVERVIEW OF YOUR ROLE:**
2. **You will play the family member** of an older man who lives in a SNF in the Seattle area. You are the legal health care decision-maker for the patient.
3. **Wait outside the room** until the learners are ready to disclose the event to you (about 30 minutes).
4. **There will be four “teams” who will disclose the same error to you**. You will pretend to be hearing it for the first time with the first three groups. The instructions provide three twists to assist you to change your response for each team to keep the experience fresh for the learners. The fourth team will present a dental “twist” to the case.

1. **Each team will have 10 minutes to disclose the error to you**. Help to keep them on time by bringing the conversation to a close by suggesting that you need time to think and ask if it would be possible to talk again if you have more questions.
2. **After each team’s disclosure**, the facilitator will quickly debrief each team (5 min). During this time, it is best to leave the room and re-enter with the next team. If you do not leave the room, look down and do not react to the team’s comments. Do not engage with them at this point. Stay in character.
3. **General debrief**: After the last team’s disclosure and quick debrief, the whole group will debrief, starting with comments from you. Stay in role for your initial comments before introducing yourself by your real name and professional role.
4. **Your comments to learners**: What you say to the learners will make a lasting impression! **Emphasize what learners did well**. **Minimize critical comments.**
   - Tie specific things said by them to your increased understanding of the error
   - Don’t tell them ‘you should have said’—let them figure this out
   - Rather than say you didn’t like what they said, tell them the effect on you as a family member—you were confused, you were irritated, you didn’t understand, you wondered whether people were being truthful, etc.
   - Be conscious of giving feedback to someone from all professional groups on the teams. Try not to focus all your comments on one professional group.
5. **More ideas for comments**: See “Error Disclosure: Potential Learning Pearls” and “Interprofessional Teams: Potential Learning Pearls”

**II. DETAILED CASE DESCRIPTION FOR ACTOR**

Background: Albert, ALBERT JACKSON, is 92, and lives in a nearby skilled nursing facility (SNF) facility because he has become progressively confused over the past five years. Albert still recognizes you but usually is confused about where he is, the date, etc. He needs help with showering, eating, dressing, etc. You worry about how quickly Albert will continue to decline. You also wonder when the confusion and dependency will get to the point where Albert would not want life-sustaining treatment anymore. Albert has not been hospitalized for two years. However, about a year ago he had a minor infection that required antibiotics and he received penicillin pills in the skilled nursing facility. He developed hives, an itchy mouth and abdominal cramping indicating a drug allergy. He recovered without problems. Since then he has had an allergy alert for penicillin that is noted in his SNF chart and as a special armband.

Two days ago you got a call from the SNF that Albert was refusing to eat. The SNF caregivers were concerned that he might be experiencing dental pain. With your permission, they scheduled a dental examination with Albert’s regular dentist.

Yesterday at 1 pm Albert had a dental exam. The dentist found no urgent dental problems. You were not able to take Albert to the appointment but a paid caretaker who is available for these appointments accompanied him. The dentist called you to say that Albert would benefit from a cleaning sometime in the next six months but that there were no signs of abscess or other sources of dental pain. S/he said that in all likelihood Albert’s reluctance to eat was part of his worsening dementia.

At 8 pm you got a call from the SNF that Albert had a fever and the SNF staff suspected pneumonia. You agreed an ambulance should be called. Albert was taken to the ED and then ended up in the ICU. You are worried about why Albert is so sick. You came to the hospital late last night and saw Albert in the ICU. He had a tube in his mouth that the nursing staff said was helping him breathe. It looked very uncomfortable. He barely opened his eyes and didn’t seem to recognize you at all.

This morning you returned to the hospital. Albert is still in the ICU but the breathing tube is out of his mouth. He is on some oxygen with nasal prongs, has an IV and is getting antibiotics through the IV. He looks better but he still does not recognize you. You are going to talk with the team soon to learn more.

Character background: You and Albert were close growing up. You grew up in Seattle as an only child. After your mom passed away from breast cancer, you and Albert became even closer. He lived alone for ten years until he started becoming increasingly forgetful and confused over about five years. You were relieved when Albert agreed to move into the SNF because you had begun to fear for his safety. Albert has declined steadily and went from independent living to assisted living to the nursing home unit over three short years. Now Albert is increasingly frail and confused about where he is living and dates/times. However, he always recognizes you and appreciates your visits several times a week.

ONE WEEK LATER: After Albert is discharged from the hospital, you make an appointment with the dentist that saw him the same day he was brought to the ED with acute pneumonia. The dental team has received the hospital discharge summary noting that he was admitted with acute pneumonia, had an allergic reaction to pipercillin/tazobactam, and required intubation and ICU care briefly. Albert recovered uneventfully from the allergic reaction and pneumonia and was discharged after 5 days in the hospital. He has returned to the SNF and is now eating and drinking again. He also recognizes you, much to your relief. You are confused about how Albert could have been seen at the dental clinic less than 8 hours before he was taken to the ED. You wonder why the emerging signs of pneumonia were not picked up. You will talk with the dentist/s at the dental clinic one week after the original event.

1. **ACTOR INSTRUCTIONS**
2. **Start the scenario:** You will be worried and anxious about Albert’s health. Your emotional level should be a 2 or 3 out of 10. There are three variations for three ED-based teams and a fourth variation for a dental team:

- First ED team: Focus **on your worry about why Albert does not recognize you**. Start with a statement such as, *“I am so relieved to talk with you! I’ve been worried about Albert. I hope this wasn’t a stroke. Has Albert taken a turn for the worse?”*
- Second ED team: **Focus on your irritation that you have been kept waiting so long to hear what really happened**. Start the interaction with a statement such as *“Finally! I have been here for an hour waiting to talk. I need to know what happened to Albert.”*
- Third ED team: **Focus on your gratitude for Albert’s care and relief that he is alive.** Start the interaction with a statement such as, *“I am so glad to see you, thank you so much for taking this time to talk with me and for saving Albert’s life.”*
- Dental team: **Focus on your appreciation that the dentist(s) have taken time to meet with you so that you can better understand what happened.** *“I really appreciate you talking to me – you’ve taken care of Albert for a long time. You got the discharge summary from the hospital? I just keep wondering if there is something we could have done to prevent Albert from getting as sick as he did. You saw him that day when he wouldn’t eat – what do you think?”*

1. **When you discover the error:** Keep asking questions about what happened to Albert, until you learn that it was an error that led to his ICU admission. EG: “*Why he is so sick that he is in the ICU? He’s had pneumonia before and never ended up in the ICU?” “He didn’t sound this sick when the SNF called?”*

- First ED team: When you realize this was an error, you will be relieved that it is not something “permanent”.

- Second ED team: When you realize this was an error, you will share that you were suspicious it was something that wasn’t supposed to happen by the way people kept telling you “the team would be by to talk with you”.
- Third ED team: When you realize this was an error, express disappointment and sadness that Albert would not have been so sick if the error had not occurred.
- Dental team: **Go easy – the dental students will not have the support of an interprofessional team.** If they apologize, accept the apology and thank them.

1. **Your emotion when you discover the error:** When you realize that an error occurred and could have had even more serious effects, become **SAD**. Your emotional level should be 5 out of 10 – your greatest emotional reaction for the encounter. You might have a sharp intake of breath, put your hand to your mouth, cover your eyes in despair, look away from the team, perhaps become a bit tearful. Sadness is an inward-directed emotion – we pull into ourselves. The other person will tend to react by moving towards us (okay) or minimizing our worries/concerns (not so good). Stay SAD for a while to allow the team to respond to that emotion. If they acknowledge your emotion and stay present with it, allow yourself to become comforted. If they do not acknowledge your emotion, continue to be sad and detach from them by breaking eye contact.
2. **Ask more questions:** You should ask probing questions to try to find out what happened. If the team responds openly and appears empathetic, you can continue to probe, but with an attitude of grudging acceptance, recognizing that these people are human beings, and that mistakes do sometimes happen. If any team member appears to be evading questions or does not acknowledge your emotion, you can continue to probe, reacting with increasing frustration and mistrust of the answers you are receiving. **Keep questions directed toward finding out what happened (in the past) and away from what will happen (in the future).**
3. **(SECOND ED TEAM AND DENTAL TEAM) Get angry**: At some point in the interaction, evolve into becoming ANGRY. Your emotional level should be NO MORE THAN a 4-5 out of 10 – anger is hard to handle so don’t go overboard. Act frustrated but not personally attacking, demeaning or sarcastic. You could say something like, *“This is awful! Albert could have died! Is that what I am understanding? He may just be an old man to you, but not to me. I can’t believe this happened!”*  You might lean forward, maintain eye contact, hold silence, grit your teeth. Anger is an outward-directed emotion – we push out of ourselves. The other person will tend to react by moving back (okay) or catching our anger and becoming belligerent (not good). Hold this emotion briefly. If the team acknowledges your emotion and stays present with it, allow yourself to become comforted. If they do not acknowledge your emotion, continue to be frustrated and detach from them by crossing your arms or pushing your chair back a bit.
4. **(SECOND ED TEAM ONLY)** Blame Trigger: At some point, blame a member/s of the team for the error. Say something like, *“So you didn’t ____*[physician: check for history of allergic reaction in the chart; nurse: read the armband carefully; pharmacist: have a better system for tracking allergies).” Don’t be too harsh. Remember that the learners will be taking turns speaking so “who” you blame may shift as the learners respond. The goal is to allow the team to ‘rescue’ one member who is targeted. If the person you blame takes responsibility and apologizes, respond positively (i.e., thank them). If other team members jump in and defend, or say something like “we are all at fault”, consider saying something like, *“Well, sounds like you all messed up.”*
5. **(THIRD ED TEAM ONLY)** Trust trigger: Toward the end of interaction, ask the team how you can trust them to take care of Albert now. If they directly respond, thank them and tell them that you appreciate their honesty and will work to trust them again. Maybe tell them that you’ll be paying close attention.
6. **Help the teams wrap up**: In order to move the groups along, please help the learners get closure on the conversation after about 7-10 minutes. A good way is to suggest you need time to process the information or to talk with other family.
7. **End neutrally**. With closure, resist the urge to make the team feel good. But if the team has done well, say something like*, “I appreciate your honesty. I’m not glad this happened but I think we just need to move forward from here and I trust you to tell me when things haven’t gone right.”* If team has really floundered, consider saying something like, *“I needed to know this even though I am not happy about it. I’m glad you told me.”*

**IV: SUMMARY OF ACTOR’S EMOTIONAL RESPONSES & TRIGGERS**

|  | **First ED Team** | **Second ED Team** | **Third ED Team** | **Fourth team (Dental)** |
| --- | --- | --- | --- | --- |
| **Start** | **Focus on your worry about why Albert does not recognize you**. *“I am so relieved to talk with you! I’ve been worried about Albert. I hope this wasn’t a stroke. Has Albert taken a turn for the worse?”* | **Focus on your irritation that you have been kept waiting so long to talk with the team**. *“Finally! I have been here an hour waiting to talk with you. I need to know what happened to Albert!”* | **Start with gratitude and relief that Albert has been ‘saved’.** *“Thank you for taking such good care of Albert! I can’t believe how sick he has been. I am so grateful to you for helping him.”* | **Start with politeness that dentist has agree to meet.**  *“Thank you for meeting. I asked the hospital to send you a discharge summary – did you get that? I want to figure out what happened.”* |
| **Discovering the error** | Relieved that it is not something permanent in Albert’s decline. | Suspicious because people kept telling you *“the team will be by to talk with you”.* | Disappointed it happened. *“My poor Albert. This could have been avoided!”* | Confused the pneumonia was missed. *“I don’t get how you wouldn’t notice.”* |
| **Sad emotion** | **+++** | **+++** | **+++** | **+++** |
| **Angry emotion** |  | **+++** |  | *“It seems you could have prevented this fiasco!”* |
| **Blame trigger** |  | To MD: *“So you didn’t check for history of allergic reaction in the chart?”*  To RN: *“You didn’t read the armband carefully!?”*  To Pharm: *“You don’t track patient allergies?”*  To PA: *“Isn’t finding out about allergies your job?”* |  | **If the dental assistant is blamed, ask:** *“So you are saying it is the dental assistant’s fault?”* |
| **Trust trigger** |  |  | *“How can I trust that you won’t screw up again?”* |  |
| **Wrap-up neutrally** | **+++** | **+++** | **Also ask that the hospital discharge summary be sent to Albert’s dentist.** | **+++** |

**Error Disclosure: Learning Pearls**

1. Remember the patient: The distress of making an error can cause clinicians to lose focus on the patient. What are the medical needs of the patient right now? Also, remember the disclosure conversation is solely for the benefit of the patient and family.
2. What to tell? Treat patients and families the way you would want to be treated. Most people want to know what led to an error occurring and what will be done to ameliorate immediate and long term health consequences (“Am I okay? Am I going to be okay?”). There is no legal risk to disclosing the facts of the case as they are known at the moment, but avoid speculation. Initial impressions of how facts fit together often are wrong.
3. What else to say? In addition to understanding what happened, patients and families want to hear how similar errors will be prevented in the future. They also want to know if they will have another chance to ask additional questions. Offer explicitly to talk again.
4. Apologize – authentically: It is a skill to avoid blame in an apology and take appropriate responsibility. It is always appropriate to say, *“I am sorry this happened to you.”* When you know that you hold responsibility, the basic formula is, *“I apologize for my [action] that caused your [harm, inconvenience, worry, suffering].”* Apologize early in the conversation and don’t hesitate to apologize more than once.
5. Plan, prepare, practice, perform: Avoid “winging” these conversations to avoid being caught off guard by the patient’s questions or reactions. When that happens, our responses can appear deceptive or uncaring. Think through who will lead the disclosure, what will be said, the need to apologize, possible patient reactions, who the patient might blame and how that will be handled, etc. Patients usually expect the attending physician to lead the discussion, but in some circumstances it may be the nurse, pharmacist, PA, ARNP, etc.
6. We prefer “sad” to “mad” reactions: Sadness is perceived as an inner-directed emotion.   Clinicians move towards sadness by wanting to rescue the other person and “fix” the sadness, potentially through minimizing. Anger feels outer-directed.  Clinicians are likely to pull back, “catching” the anger and becoming irritated with the other person.  For either emotional response, clinicians need to respond to the other person’s emotion by acknowledging the emotion, communicating accountability, and apologizing for the error. *“I think if this had happened to Albert, I would also feel discouraged/angry/scared.”* The learning point is to be responsive and empathetic to both emotional presentations.
7. Blame: Patients or families may try to assign blame to a person. Teams need to think through how they will handle this to avoid abandoning that colleague (silence), having one person fall on the sword (“I’m the captain of the ship – it was my team, so my error”), or appearing to be the Keystone Cops (“We all screwed up”). We also need to avoid blaming members of the health care team who are not present. This can include other facilities, other providers, or other professions.
8. Trust: Patients and families may also tell us that they have lost trust. Explicitly address statements about trust by saying, *“We hope to rebuild your trust”* or *“We would like the opportunity to earn back your trust”.*
9. These conversations don’t end “happy”: Disclosures usually do not end with the patient or family thanking or forgiving us.  Clinicians need to not expect support from the patient.
10. Get support from your colleagues or other resources: Making an error can be one of the most devastating experiences a clinician will face. Get (and offer) support from your team members, colleagues, or other sources. Consider whether you (or your colleague) needs to go off-line temporarily for patient safety.

**Interprofessional Teams: Learning Pearls**

1. All team members feel a fiduciary relationship to the patient: Health care professionals are devastated by errors made in their care. They often believe that an error was primarily their responsibility and are surprised when they learn that others on the team feel that same strong sense of responsibility. They may not have realized that they were not solely, or even primarily, responsible for the error.
2. Balancing personal responsibility, team responsibility, and system accountability: The concept of a “Just Culture” does not mean a blame-free culture but rather one where the individual acknowledges personal accountability and the team shared responsibility while the effect of the system on errors is recognized. Teams may want to avoid blaming each other by instead blaming “the system” or another institution. Recognizing how to accept (and share) responsibility without feeling blamed is critical. Teams need to be prepared to handle the patient or family member’s attempts to fix blame on one person or group and to communicate shared responsibility.
3. Listening and speaking up are critical team skills: Listening is important but not enough for effective team communication. Good team skills require also speaking up. Team members need to contribute to discussions about patient care.
4. Providing support to each other helps teams provide better patient care: Making an error can be a devastating experience. Professionals may need to attend to the context of the error or adverse event and their emotional reaction to continue to provide safe and compassionate care to patients.
5. Role clarification: Understanding one’s own role and the roles of those in other professions, and using this knowledge appropriately, can help to establish and achieve patient goals.
6. Patient/Family-Centered Care: Teams do best when they seek out, integrate and value, as a partner, the input and engagement of the patient or family in designing and implementing care services.
7. Team functioning: Teams understand the principles of team work dynamics and group/team processes to enable effective interprofessional collaboration. For example, open discussion of any differences of opinion around what to say in the error disclosure is critical to effective disclosure.
8. Collaborative leadership: Team understands and can apply leadership principles that support a collaborative practice model. In error disclosure conversations, formal and/or informal leadership is needed to insure effective communication.
9. Good patient communication requires good team communication: Interprofessional communication among members of the team from different professions needs to be collaborative, responsive and responsible. Patients want compassionate, honest, and consistent communication with their health care professionals. To have the whole picture, teams need to communicate openly about errors and other adverse events before talking with the patient or family.

**Facilitating Interprofessional Groups: Pearls and Pitfalls**

1. One student or group of students dominates the conversation:
   - Medical students may feel confident with the learning format and know their peers
   - Nursing or PA students may have had more clinical experience
   - Pharmacy students because the error involves a drug
   - If this occurs, encourage other students to speak up: "Any thoughts on this from the perspective of a nursing/medical/PA/pharmacy student?"
2. Some learners are quiet or reluctant participants: Suggest they try something that they would like to experiment with, something that they have not tried before but would like to “have fun with”. Continue in debrief to treat their effort as an “experiment” to make the learning environment safe.
3. Personal or war stories as distractions: Learners may want to use this opportunity to bring in other examples where interprofessional interactions did not go well or where they observed another professional not behaving well. Refocus group back to the learning activity for the day and *“how we can do this well”* or *“I know we each may have had individual experiences, but today, let’s focus on this shared experience and then compare to our other experiences at the end if there is time.”*
4. Challenging comment/s from a learner: Sometimes learners say something that separates them from the other learners *(“I’m not sure we should disclose this error? Maybe it would be better to just not tell. The family won’t know if we don’t tell them.”*) If that happens, restate the comment as a **feeling** rather than a **belief** *(“I bet there are others who are feeling that they might prefer to not disclose this error. This will be hard! What are the reasons we might feel that we need to be honest as clinicians?*”). The goal is to keep all learners as a part of the learning community by allowing them to reconsider their comments in a non-judgmental way.
5. Getting sidetracked by discussions about “my profession”: Learners are likely to be genuinely interested in each other’s educational programs, normal clinical experiences, and the like. But they also may digress to avoid discussing the hard stuff – the error! Try to refocus general conversations back to the specifics of the case so learners have time for the simulation with the family member. They highly rate the opportunity for practice even though it makes them anxious.
6. Jokes: Your good humor is appreciated, but avoid attempts at interprofessional jokes. They end up a bit like Polish jokes. (Not as funny if your last name is Polanski.) The professional group being teased often ends up feeling targeted.
7. Acknowledging hierarchy/power relationships: Whatever your own professional background is, you are more likely to pay attention to those students. (We have evaluation data from prior years confirming this phenomenon.) If you are an MD acting as a facilitator, recognize that you are coming with biases. (Same for RN, PA, Pharmacist.) Try to avoid saying, *“This is the way it is”*. Instead engage in respectful curiosity or appreciative inquiry about other views *(“Tell us more about ___.” “That’s a different perspective than we’ve heard so far – thank you for sharing.”)* Demonstrate humility.
8. Biases, stereotyping, and bashing another profession: Sometimes a student will say something that is very negative about another group. For example, a nursing student might say, *“Physicians don’t learn how to communicate compassionately with patients so they just lecture AT them.”* How do you address physician, nurse, dentist, PA, or pharmacist bashing? How do you call out the stereotyping/negative communication without derailing a positive learning experience? **Our strong advice: Don’t ignore these comments.** This is a critical issue to handle as a facilitator in a non-aggressive manner. Steps:
9. Take control of the discussion briefly: *“You have brought an important issue to our discussion that I would like to take a minute to address.”*
10. Shift from the particular to the general**:** *“There are a lot of these types of beliefs – even myths – in health care. Physicians aren’t educated around communication skills. Nurses don’t learn science. Pharmacists are disengaged.”* [Try to generalize -- and perhaps soften -- what the student said].
11. Reaffirm the purpose of IPE learning**:** *“We want to offer all of you this opportunity to learn together, something most of us in health care did not have, so that you have the opportunity to not perpetuate those myths and stereotypes.”*
12. Thank the student for his/her comment**:** *“Thank you for saying that. I know most of us probably have beliefs about each other’s professions and training that we are bringing to this experience. We hope that you can use this experience to explore those beliefs. It will help if we do that in a tone of respectful curiosity. So one idea would be to ask your colleagues questions about the stereotypes you might hold to give your team* a chance to discuss. Another idea is to observe. See if the stereotype seems accurate.”
13. Ask for the group’s willingness to discuss hard issues: *“Does that sound reasonable? Would you all be willing to have this be a place where we can tackle this type of question if it arises – as a team?”*
14. Re-engage learners in the current topic of discussion: Ask a student from a “neutral” profession to talk about his/her professional training or role relative to the topic. For example, *“As a pharmacy student, what type of communication training do you get to assist you with effectively and compassionately counseling patients?”*

**Additional resources: Small group skills practice**

Back AL, Arnold RM, Tulsky JA, Baile WF, Edwards K. "Could I add something?": Teaching communication by intervening in real time during a clinical encounter. Acad Med. 2010 Jun;85(6):1048-51. PMID: 20505408; PubMed Central PMCID: PMC3097516.

Back AL, Arnold RM, Baile WF, Tulsky JA, Fryer-Edwards K. Humanism in oncology. What makes education in communication transformative? J Cancer Educ. 2009;24(2):160-2. PubMed PMID: 19431035; PubMed Central PMCID: PMC2697957.

Jackson VA, Back AL. Teaching communication skills using role-play: an experience-based guide for educators. J Palliat Med. 2011 Jun;14(6):775-80. PubMed PMID: 21651366; PubMed Central PMCID: PMC3155105.

**Additional Resources: Error Disclosure**

Gallagher TH. A 62-year-old woman with skin cancer who experienced wrong-site surgery: review of medical error. JAMA. 2009 Aug 12;302(6):669-77. PubMed PMID: 19584321.

Gallagher TH, Studdert D, Levinson W. Disclosing harmful medical errors to patients. N Engl J Med. 2007 Jun 28;356(26):2713-9. PubMed PMID:17596606.

Garbutt J, Waterman AD, Kapp JM, Dunagan WC, Levinson W, Fraser V, Gallagher TH. Lost opportunities: how physicians communicate about medical errors. Health Aff (Millwood). 2008 Jan-Feb;27(1):246-55. PubMed PMID: 18180501.

Mastroianni AC, Mello MM, Sommer S, Hardy M, Gallagher TH. The flaws in state 'apology' and 'disclosure' laws dilute their intended impact on malpractice suits. Health Aff (Millwood). 2010 Sep;29(9):1611-9. PubMed PMID: 20820016.

Shannon SE, Foglia MB, Hardy M, Gallagher TH. Disclosing errors to patients: perspectives of registered nurses. Jt Comm J Qual Patient Saf. 2009 Jan;35(1):5-12. PubMed PMID: 19213295.

White AA, Bell SK, Krauss MJ, Garbutt J, Dunagan WC, Fraser VJ, Levinson W, Larson EB, Gallagher TH. How trainees would disclose medical errors: educational implications for training programmes. Med Educ. 2011 Apr;45(4):372-80. PubMed PMID: 21401685; PubMed Central PMCID: PMC3501535.

Wu AW, Huang IC, Stokes S, Pronovost PJ. Disclosing medical errors to patients: it's not what you say, it's what they hear. J Gen Intern Med. 2009 Sep;24(9):1012-7. PubMed PMID: 19578819; PubMed Central PMCID: PMC2726881.
