# Supplementary material for: Interprofessional Error Disclosure Training for Medical, Nursing, Pharmacy, Dental, and Physician Assistant Students
Source: MedEdPORTAL. 2017 Jul 21;13:10606. doi: 10.15766/mep_2374-8265.10606 (PMC6338166; doi:10.15766/mep_2374-8265.10606)
Supplement: Supplementary file 1 — A. Interprofessional Error Disclosure Module folder B. Error Disclosure Faculty Facilitators Guide.docx C. Profession-Specific Cases.docx D. Error Disclosure Pocket Cards.pdf E. Error Disclosure Slides.pptx [file mep-13-10606-s001.zip › C. Profession-Specific Cases.docx]

**Piperacillin/Tazobactam (Zosyn) Case: PHYSICIAN**

Background: ALBERT JACKSON, 92 y.o. male patient, was admitted to emergency department (ED) from local skilled nursing facility (SNF). Before admission, he became increasing SOB, with yellow-greenish sputum, and febrile to 38.5 with pulse oximetry = 88. The SNF staff were concerned the patient had developed pneumonia and, after receiving permission from the patient’s family member, called an ambulance for transport to the ED.

### Admission Status: You were involved in the aftermath of a difficult, and ultimately unsuccessful resuscitation attempt when Mr. Jackson arrived so other personnel did the initial physical exam of Mr. Jackson and got a chest x-ray, and labs. You picked up the case at that point and reviewed the results, reexamining Mr. Jackson to confirm the obvious diagnosis of pneumonia. You started him on Zosyn (Piperacillin/Tazobactam) IV every 6 hours. First dose was given at 2200. He experienced an anaphylactic reaction within 20 minutes involving flushing, itching, difficulty swallowing, coughing, wheezing, hypotension and difficulty breathing. Patient was intubated and treated with epinephrine. He was transferred to the ICU for close observation around 2330 with orders for additional epinephrine as needed, discontinue piperacillin/tazobactam and start vancomycin and levofloxacin IV.

### Current: Mr. Jackson was extubated this AM without incident. Currently he is afebrile, vital signs stable, rash resolving. He is able to follow commands, but is confused to person, place and time. Notes from the SNF indicate that the patient has required assistance with ADLs for approximately 3 years due to confusion and general physical deterioration and weakness. He generally recognizes family members but is confused to time and place.

### This morning you learn that Mr. Jackson had a penicillin allergy documented in the SNF records that accompanied him. There was a notation about a penicillin allergy from one-year prior when the patient received oral penicillin in the SNF (developed hives and abdominal cramping). Piperacillin/tazobactam is contra-indicated with penicillin allergies. Albert’s last hospitalization was two years prior. The penicillin allergy was also noted on a face page but this page was out of order in the record and you had not seen the allergy on your initial review. In addition, the allergy was not noted in your facility’s computer system presumably because it occurred since the last admission. There was an alert for another allergy (codeine) in the computer system.

### Situation: Albert’s family member, who is also his medical power of attorney, is here visiting. You and the other members of the health care team are going to talk with this person about the events of the evening and why Albert is in the ICU.

**Piperacillin/Tazobactm (Zosyn) Case: NURSE**

Background: ALBERT JACKSON is a 92 y.o. male patient, admitted to the ED from a local skilled nursing facility (SNF) around 1800. He became increasing SOB with yellow-greenish sputum and was febrile to 38.5 with pulse oximetry = 88 yesterday. The SNF staff were concerned about pneumonia and, after receiving permission from patient’s family member, called an ambulance for transport to ED.

### Admission Status: You helped admit Albert in the ED. It was difficult to examine him because he moaned when uncovered. You helped get a chest x-ray, labs and do an initial assessment. Everything confirmed pneumonia. The physician ordered Zosyn (Piperacillin/Tazobactam) IV every 6 hours. You gave the first dose at 2200 and within 20 minutes Albert experienced an anaphylactic reaction involving flushing, itching, difficulty swallowing, coughing, wheezing, hypotension and dyspnea. With his already compromised pulmonary status, Albert needed to be intubated. He responded well to epinephrine.

### Albert was to remain on the ventilator for a few hours to protect his airway until the allergic reaction resolved. He was transferred to the ICU around 2330 with orders for additional epinephrine as needed, discontinue piperacillin/tazobactam and start vancomycin and levofloxacin IV.

### Current: This morning you called the ICU to check on Albert and learned that he was extubated early this AM. He is currently afebrile, vital signs stable, rash resolving, on some oxygen with pulse oximetry = 97. He is following commands but remains confused to person, place and time. The SNF reported that Albert required assistance with ADLs due to his confusion and general weakness but always recognized his family. His family have visited Albert twice in the ICU but he does not recognize them yet.

### When talking with the ICU, you learn that the documentation from the SNF included information about a penicillin allergy. Piperacillin/tazobactam is contra-indicated with penicillin allergies. The ICU nurse also asks if you had noticed the allergy armband. You are stunned. You go up to the ICU to look again at the armbands. You remember glancing at them when you admitted Albert but they were both difficult to read because they had gotten wet numerous times. When you look at them again, you can now read “penicillin”. You check Albert’s chart and see that he had an incident one-year prior in the SNF when he received oral penicillin and developed hives and abdominal cramping. Albert’s last hospitalization was two years prior. You had not noticed this on your initial record review.

### Situation: Albert’s family member is here visiting and would like to talk with the team about the events of the evening and why Albert is in the ICU. The physician and PA you worked with in the ED yesterday evening are here also.

**Piperacillin/Tazobactam (Zosyn) Case: PHARMACIST**

Background: ALBERT JACKSON, 92 y.o. male patient, admitted to ED from local skilled nursing facility (SNF) around 1800. Patient had SOB with yellow-greenish sputum and febrile to 38.5 with pulse oximetry = 88. SNF staff were concerned about pneumonia and, after receiving permission from patient’s DPAHC, called ambulance for transport to ED.

### Admission Status: Patient received chest x-ray, labs and physical exam in ED confirming diagnosis of healthcare-associated pneumonia. Started on Zosyn (piperacillin sodium/ tazobactam sodium) IV every 6 hours. His first dose was given at 2200 and the patient experienced an anaphylactic reaction within 20 minutes involving flushing, itching, difficulty swallowing, coughing, wheezing, hypotension and difficulty breathing. He was intubated and treated with epinephrine IM.

### The patient was transferred to the ICU for close observation around 2330 with orders for additional epinephrine as needed, discontinue piperacillin/tazobactam, and start vancomycin and levofloxacin IV.

### Current: Patient was extubated from ventilator early in AM without incident. Currently is afebrile, vital signs stable, rash resolving. He is following commands, but remains confused to person, place and time.

### When you got to work this morning you learn from the pharmacist covering the ICU that there was a penicillin allergy noted in Albert Jackson’s chart that was apparently missed by the team. Piperacillin/tazobactam is contra-indicated in patients with a known penicillin allergy. You do not remember looking at the records that accompanied the patient but did carefully review the patient’s computerized hospital record and the only allergy listed was a codeine allergy.

### You went to the ICU to look at the record yourself. There was a notation about a penicillin allergy from one year prior when the patient received oral penicillin in the SNF (he developed hives and abdominal cramping). Albert’s last hospitalization was two years prior. You do not know why the rest of the team did not see this and now regret that you did not review these records. You also read that the patient has lived in the SNF for over three years due to increased confusion and need for assistance with activities of daily living (showering, feeding, etc.) He has had a progressive decline. He has a family member who is his durable power of attorney for health care decisions. From the chart, this person appears to be very involved in the patient’s care and visits frequently.

### Situation: Albert’s family member is here visiting. The physician and nurse from the ED who admitted Albert are also in the ICU, apparently also having learned of the penicillin allergy and were also reviewing the medical record. You learn that the family has asked to talk with the team about the events of the evening and why Albert is in the ICU.

**Piperacillin/Tazobactam (Zosyn Case): PHYSICIAN ASSISTANT**

Background: ALBERT JACKSON, 92 y.o. male patient, was admitted to emergency department (ED) from local skilled nursing facility (SNF). Before admission, he became increasing SOB, with yellow-greenish sputum, and febrile to 38.5 with pulse oximetry = 88. The SNF staff were concerned the patient had developed pneumonia and, after receiving permission from the Albert’s family member, called an ambulance for transport to the ED.

### Admission Status: You did the intake examination for Mr. Jackson and took report from the ambulance crew. Mr. Jackson received a chest x-ray, labs and physical exam in ED confirming diagnosis of pneumonia. You reported the information to the physician on duty when he/she finished with a prior case. The physician started Mr. Jackson on Zosyn (Piperacillin/ Tazobactam) IV every 6 hours. First dose was given at 2200. Mr. Jackson experienced an anaphylactic reaction within 20 minutes involving flushing, itching, difficulty swallowing, coughing, wheezing, hypotension and difficulty breathing. Patient was intubated and treated with epinephrine.

### Mr. Jackson was transferred to the ICU for close observation around 2330 with orders for additional epinephrine as needed, discontinue piperacillin/tazobactam and start vancomycin and levofloxacin IV.

### Current: Mr. Jackson was extubated this AM without incident. Currently he is afebrile, vital signs stable, rash resolving. He is able to follow commands, but is confused to person, place and time. Notes from the SNF indicate that the patient has required assistance with ADLs for approximately 3 years due to confusion and general physical deterioration and weakness. He usually recognizes family members but is confused to time and place.

### This morning you learn that Mr. Jackson had a penicillin allergy documented in the SNF records that accompanied him. There was a notation about a penicillin allergy from one-year prior when the patient received oral penicillin in the SNF (developed hives and abdominal cramping). Piperacillin/tazobactam is contra-indicated with penicillin allergies. Albert’s last hospitalization was two years prior. The penicillin allergy was also noted on a face page but this page was out of order in the record and you had not seen the allergy on your initial review. In addition, the allergy was not noted in your facility’s computer system presumably because it occurred since the last admission. There was an alert for another allergy (codeine) in the computer system. You vaguely remember the ambulance crew saying something about allergies but you had just come from a very complicated code and remember struggling with feeling distracted when you took report from them.

### Situation: Albert’s family member is here visiting. The physician has asked you to join the team to talk with the family about the events of the evening and why Albert is in the ICU.

**Piperacillin/Tazobactam (Zosyn Case): HOSPITAL ADMINISTRATOR or RISK MANAGER**

Background: ALBERT JACKSON, a 92 y.o. male patient, was admitted to emergency department (ED) from a local skilled nursing facility. Just prior to admission, he became increasing SOB, with yellow-greenish sputum, and febrile to 38.5 with pulse oximetry = 88. The facility staff were concerned the patient had developed pneumonia and, after receiving permission from his family member, called an ambulance for transport to the ED.

### Admission Status: The ED team took report from the ambulance crew. Mr. Jackson received a chest x-ray, labs and physical exam in ED confirming diagnosis of pneumonia and was started on Zosyn (Piperacillin/Tazobactam) IV. The first dose was given at 2200 (10 PM). Mr. Jackson experienced an anaphylactic reaction within 20 minutes involving flushing, itching, difficulty swallowing, coughing, wheezing, hypotension and difficulty breathing. He was intubated, placed on a ventilator, treated with epinephrine and transferred to the ICU for close observation. The piperacillin/tazobactam was discontinued and vancomycin and levofloxacin were started.

### Current: Mr. Jackson was extubated this AM without incident. Currently he is recovering from both the anaphylaxis and pneumonia. He is able to follow commands, but is confused to person, place and time. Notes from the SNF indicate that he was able to recognize family members before this admission though he was confused to place and time.

### This morning you learned that Mr. Jackson had a penicillin allergy documented in the SNF records that accompanied him to the ED. There was a notation about a penicillin allergy from one-year prior when the patient received oral penicillin in the SNF (developed hives and abdominal cramping). Piperacillin/tazobactam is contra-indicated with penicillin allergies. Albert’s last hospitalization was two years prior. The penicillin allergy was also noted on the medical record face page. The allergy was not noted in your facility’s computer system presumably because it occurred since the last admission. There was an alert for another allergy (codeine) in the computer system.

### Situation: Albert’s family member is here visiting. The health care team is planning to meet with the family to discuss what happened. You are going to join the team as they plan for their meeting with the family about the events of the evening and why Albert is in the ICU.

**Piperacillin/Tazobactam (Zosyn) Case: Dentist**

Background: ALBERT JACKSON is a 92 y.o. man who lives in a local skilled nursing facility (SNF). You have seen him regularly over the past five years for his dental care. Mr. Jackson steadily has declined from dementia. At his last visit, you noted that he seemed confused as to where he was and what was happening, but clearly recognized the family member who had accompanied him to the appointment.

One week ago: Mr. Jackson was seen in your clinic because the caregivers at his SNF had noticed a 2-3 day pattern of decreased appetite and unwillingness to have food in his mouth. These behaviors had raised a suspicion of dental pain. A paid assistant accompanied him to this appointment as his family member had work obligations. Albert seemed more subdued than the last time you saw him. As per clinic protocol, the dental hygienist took Mr. Jackson’s vital signs and noted them on the clinic record (HR 115, BP 110/62, RR 28, T 37.4). In the past Albert has been reasonably cooperative with oral exams. This time he was reluctant to lay back in the dental chair and was more difficult to examine because he insisted on breathing through pursed lips. You found no new or acute dental problems, no signs of oral infection (e.g., abscess), and no obvious sources of dental pain. Your assessment was that Albert’s increasing refusal to eat was most likely associated with progressing dementia.

Yesterday: You received a discharge summary note from the local hospital regarding Mr. Jackson. You learn that six hours after your evaluation of him for dental pain, he was taken to the emergency department of the local hospital due to increasing shortness of breath, yellow-greenish sputum, and febrile (38.5° C). He was diagnosed with acute pneumonia and treated with Zosyn (Piperacillin/Tazobactam) IV in the ED. Mr. Jackson experienced an anaphylactic reaction within 20 minutes involving flushing, itching, difficulty swallowing, coughing, wheezing, hypotension and difficulty breathing. He required epinephrine, intubation, (for approximately 14 hours) and ICU care for two days. Piperacillin/tazobactam was discontinued and vancomycin and levofloxacin were started. Mr. Jackson was discharged back to his SNF after a five-day hospitalization on oral antibiotics.

This morning: Mr. Jackson’s family member called this morning asking if you had received the hospital discharge summary and requesting to meet with you briefly to discuss, “what happened last week” when Albert was seen in your clinic. You have open time in your schedule and are preparing to meet Mr. Jackson’s family member (who is also his durable power of attorney for health care). In looking at your notes from that visit, you realize his vital signs were abnormal. You don’t recall having been concerned about Albert’s vitals at the time. He seemed to be breathing comfortably at the time of the exam, but now you regret not having picked up on the clues to his acute illness.
