# Supplementary material for: Interprofessional Error Disclosure Training for Medical, Nursing, Pharmacy, Dental, and Physician Assistant Students
Source: MedEdPORTAL. 2017 Jul 21;13:10606. doi: 10.15766/mep_2374-8265.10606 (PMC6338166; doi:10.15766/mep_2374-8265.10606)
Supplement: Supplementary file 1 — A. Interprofessional Error Disclosure Module folder B. Error Disclosure Faculty Facilitators Guide.docx C. Profession-Specific Cases.docx D. Error Disclosure Pocket Cards.pdf E. Error Disclosure Slides.pptx [file mep-13-10606-s001.zip › D. Error Disclosure Pocket Cards.pdf]

Key Steps in Team Error Disclosure

Key Step	Target Behaviors
Team Discusses the Error	1. Acknowledges error<br>2. Conducts blame-free communication during team conversation<br>3. Demonstrates team-oriented communication<br>4. Negotiates differences of opinion collaboratively<br>5. Recognize emotional impact of errors on team members
Team Plans the Disclosure	1. Advocates for full disclosure<br>2. Plans roles for disclosure<br>3. Anticipate patient's questions and reactions<br>4. Plans responses to patient
Team Discloses Error to Patient	1. Conducts explicit disclosure of error to patient<br>2. Responds forthrightly to patient questions about event<br>3. Apologizes upfront and early in conversation<br>4. Conducts blame-free disclosure, acknowledges personal role<br>5. Offers plans to prevent future errors<br>6. Plans follow up with patient

Key Steps in Team Error Disclosure

Key Step	Target Behaviors
Team Discusses the Error	1. Acknowledges error<br>2. Conducts blame-free communication during team conversation<br>3. Demonstrates team-oriented communication<br>4. Negotiates differences of opinion collaboratively<br>5. Recognize emotional impact of errors on team members
Team Plans the Disclosure	1. Advocates for full disclosure<br>2. Plans roles for disclosure<br>3. Anticipate patient's questions and reactions<br>4. Plans responses to patient
Team Discloses Error to Patient	1. Conducts explicit disclosure of error to patient<br>2. Responds forthrightly to patient questions about event<br>3. Apologizes upfront and early in conversation<br>4. Conducts blame-free disclosure, acknowledges personal role<br>5. Offers plans to prevent future errors<br>6. Plans follow up with patient

Key Steps in Team Error Disclosure

Key Step	Target Behaviors
Team Discusses the Error	1. Acknowledges error<br>2. Conducts blame-free communication during team conversation<br>3. Demonstrates team-oriented communication<br>4. Negotiates differences of opinion collaboratively<br>5. Recognize emotional impact of errors on team members
Team Plans the Disclosure	1. Advocates for full disclosure<br>2. Plans roles for disclosure<br>3. Anticipate patient's questions and reactions<br>4. Plans responses to patient
Team Discloses Error to Patient	1. Conducts explicit disclosure of error to patient<br>2. Responds forthrightly to patient questions about event<br>3. Apologizes upfront and early in conversation<br>4. Conducts blame-free disclosure, acknowledges personal role<br>5. Offers plans to prevent future errors<br>6. Plans follow up with patient

Key Steps in Team Error Disclosure

Key Step	Target Behaviors
Team Discusses the Error	1. Acknowledges error<br>2. Conducts blame-free communication during team conversation<br>3. Demonstrates team-oriented communication<br>4. Negotiates differences of opinion collaboratively<br>5. Recognize emotional impact of errors on team members
Team Plans the Disclosure	1. Advocates for full disclosure<br>2. Plans roles for disclosure<br>3. Anticipate patient's questions and reactions<br>4. Plans responses to patient
Team Discloses Error to Patient	1. Conducts explicit disclosure of error to patient<br>2. Responds forthrightly to patient questions about event<br>3. Apologizes upfront and early in conversation<br>4. Conducts blame-free disclosure, acknowledges personal role<br>5. Offers plans to prevent future errors<br>6. Plans follow up with patient
