# Supplementary material for: Interprofessional Error Disclosure Training for Medical, Nursing, Pharmacy, Dental, and Physician Assistant Students
Source: MedEdPORTAL. 2017 Jul 21;13:10606. doi: 10.15766/mep_2374-8265.10606 (PMC6338166; doi:10.15766/mep_2374-8265.10606)
Supplement: Supplementary file 1 — A. Interprofessional Error Disclosure Module folder B. Error Disclosure Faculty Facilitators Guide.docx C. Profession-Specific Cases.docx D. Error Disclosure Pocket Cards.pdf E. Error Disclosure Slides.pptx [file mep-13-10606-s001.zip › E. Error Disclosure Slides.pptx]

## Slide 1
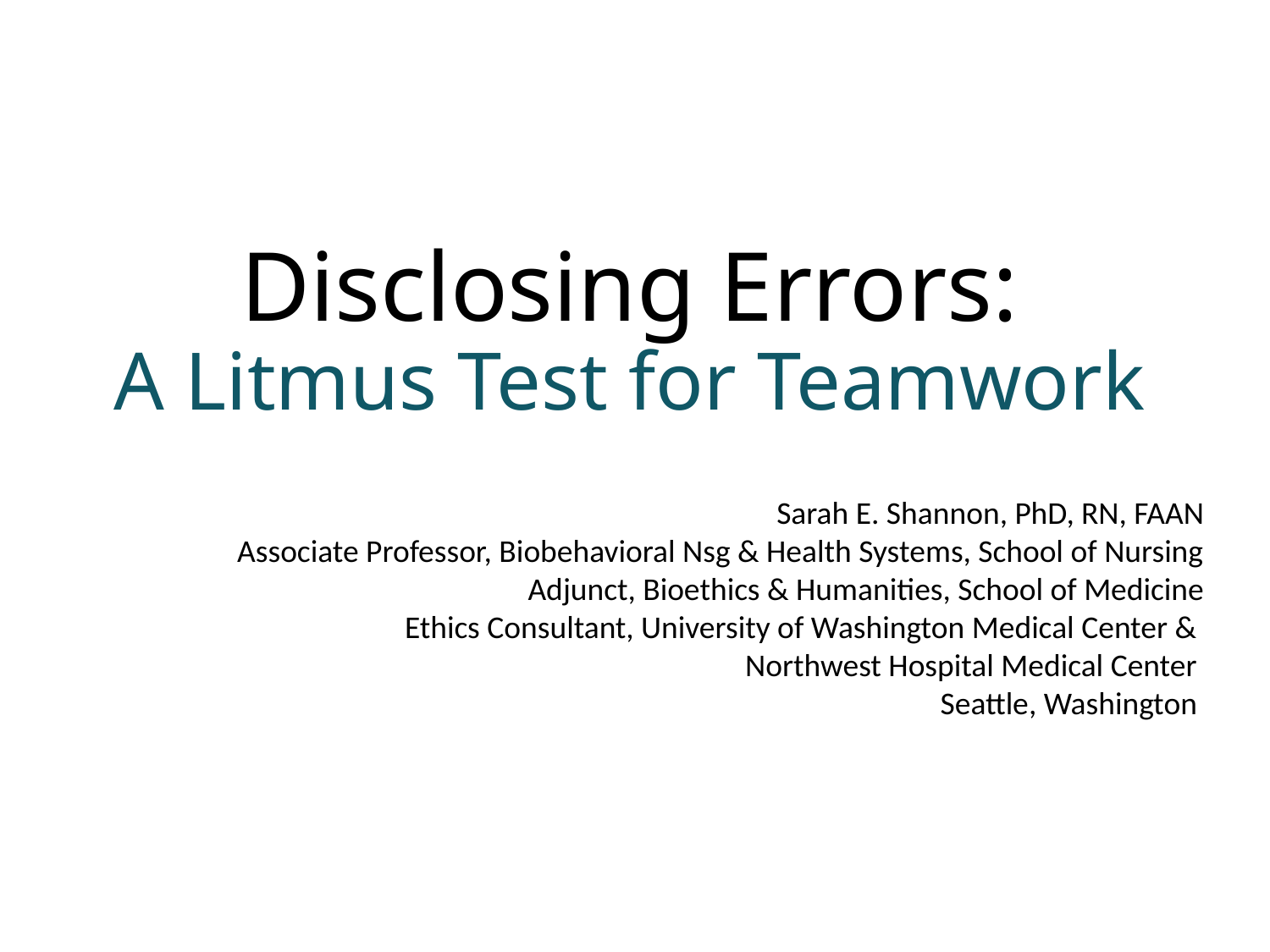

# Disclosing Errors: A Litmus Test for Teamwork
Sarah E. Shannon, PhD, RN, FAAN
Associate Professor, Biobehavioral Nsg & Health Systems, School of Nursing
Adjunct, Bioethics & Humanities, School of Medicine
Ethics Consultant, University of Washington Medical Center &
Northwest Hospital Medical Center
Seattle, Washington

## Slide 2
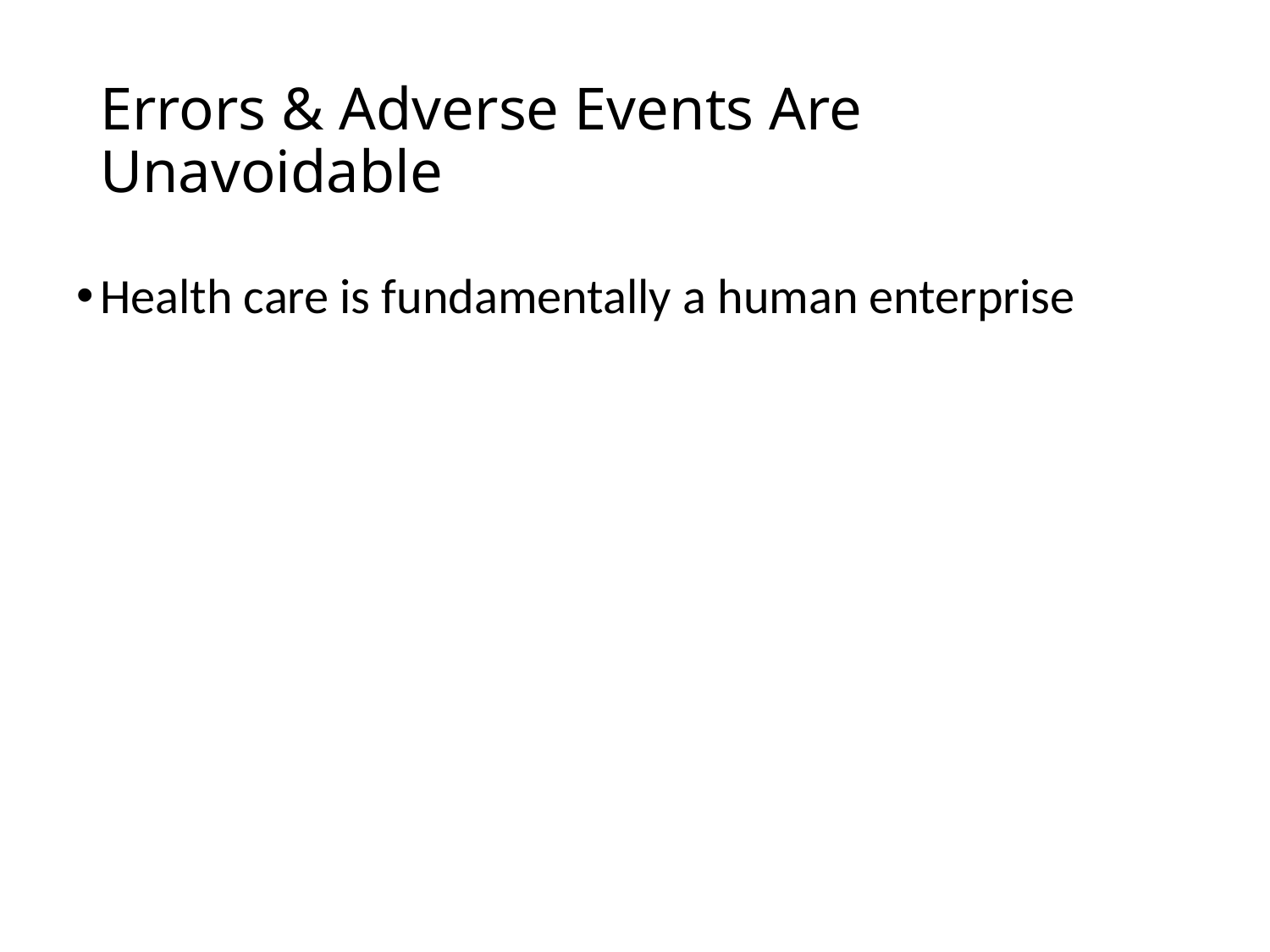

# Errors & Adverse Events Are Unavoidable
Health care is fundamentally a human enterprise

## Slide 3
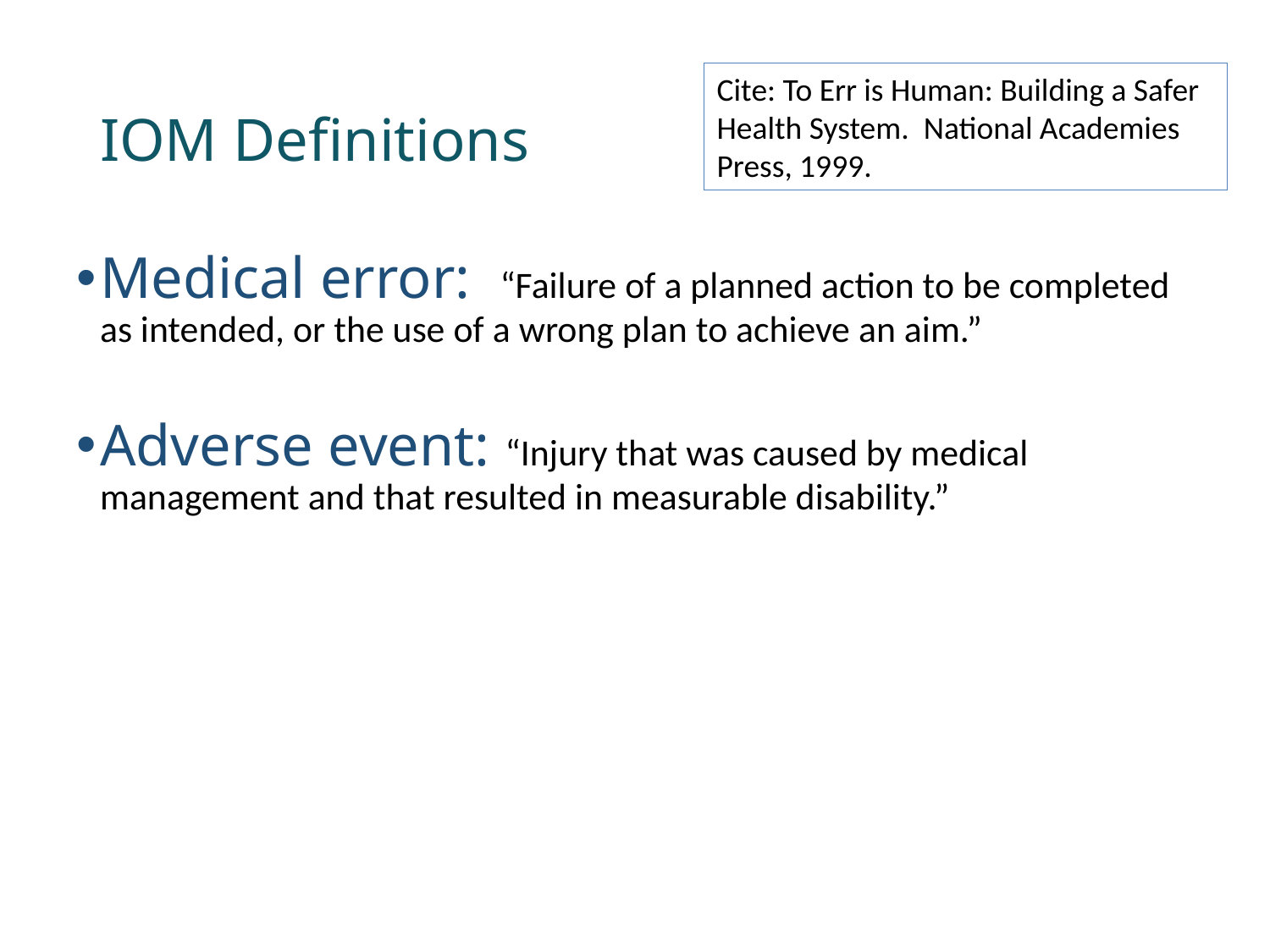

# IOM Definitions
Cite: To Err is Human: Building a Safer Health System. National Academies Press, 1999.
Medical error: “Failure of a planned action to be completed as intended, or the use of a wrong plan to achieve an aim.”
Adverse event: “Injury that was caused by medical management and that resulted in measurable disability.”

## Slide 4
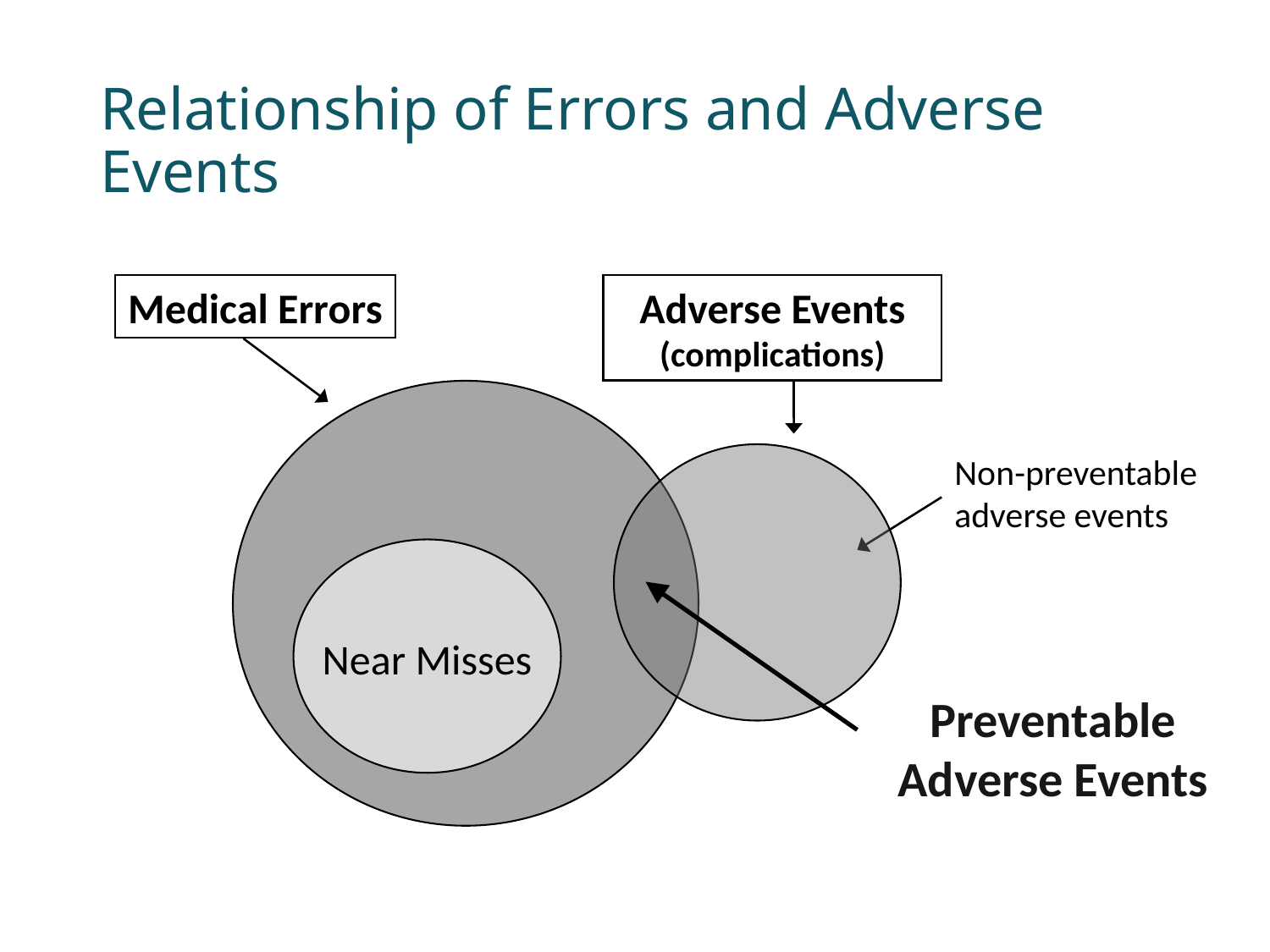

# Relationship of Errors and Adverse Events
Medical Errors
Adverse Events (complications)
Non-preventable adverse events
Near Misses
Preventable Adverse Events

## Slide 5
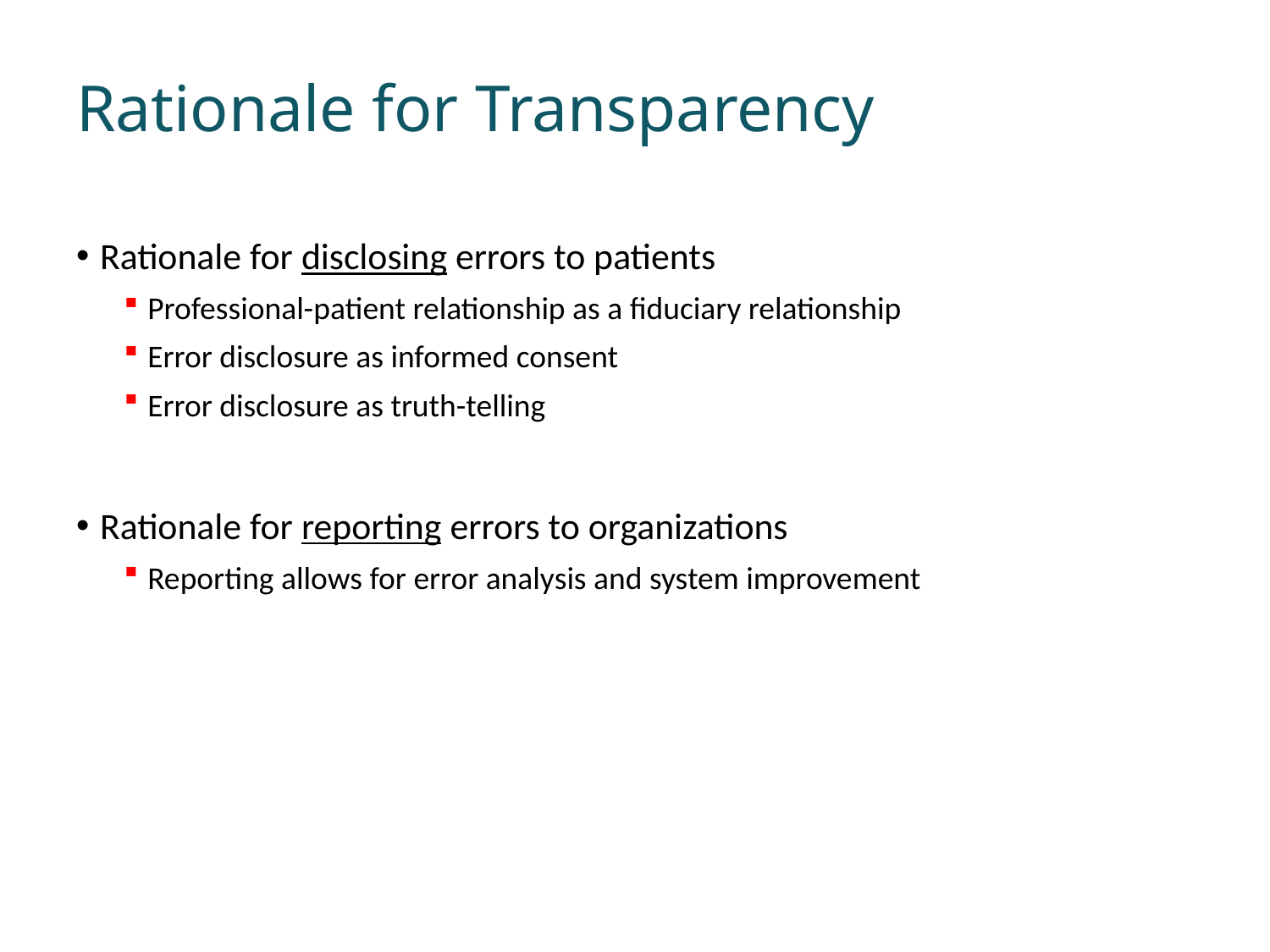

# Rationale for Transparency
Rationale for disclosing errors to patients
Professional-patient relationship as a fiduciary relationship
Error disclosure as informed consent
Error disclosure as truth-telling
Rationale for reporting errors to organizations
Reporting allows for error analysis and system improvement

## Slide 6
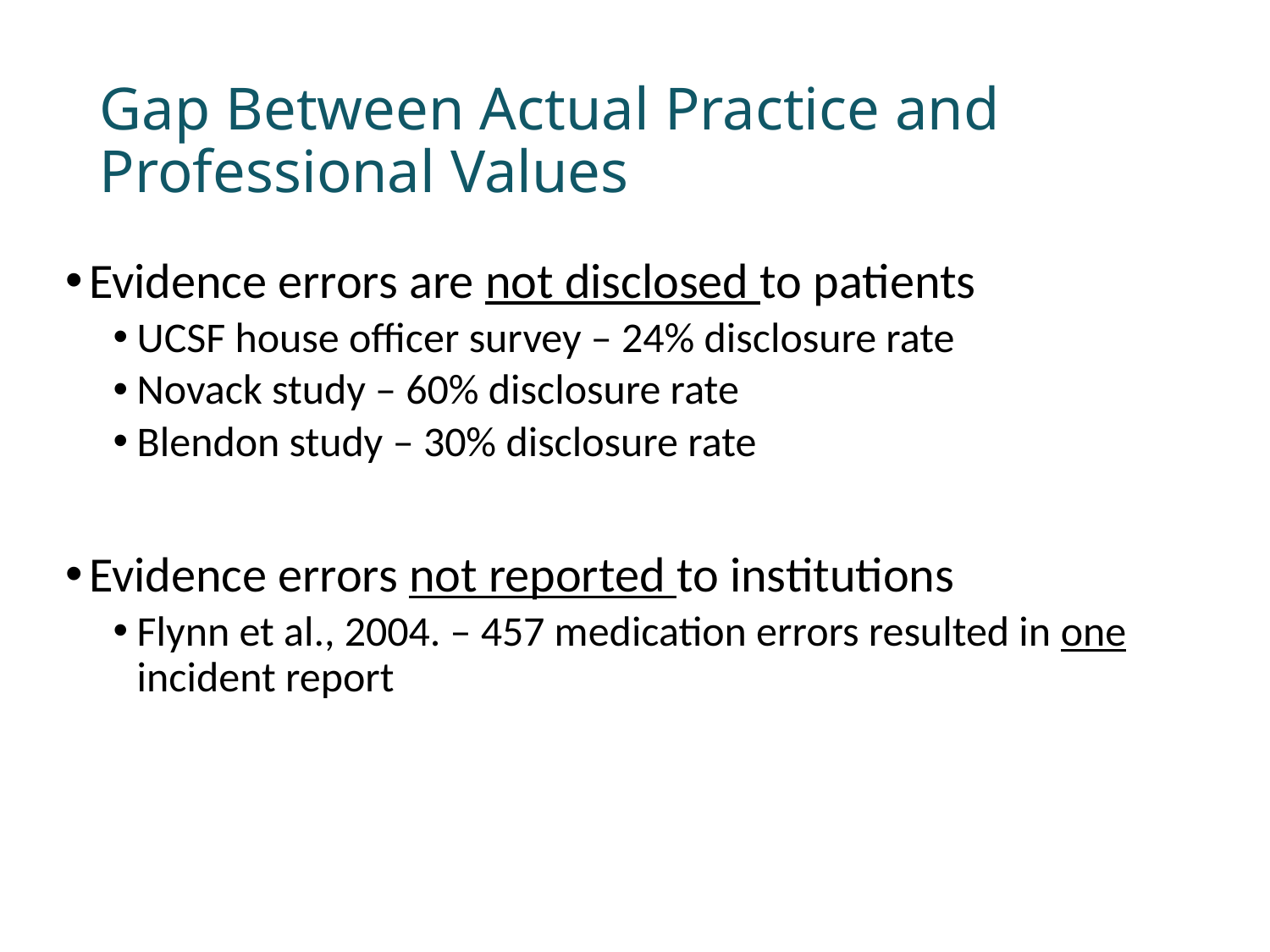

# Gap Between Actual Practice and Professional Values
Evidence errors are not disclosed to patients
UCSF house officer survey – 24% disclosure rate
Novack study – 60% disclosure rate
Blendon study – 30% disclosure rate
Evidence errors not reported to institutions
Flynn et al., 2004. – 457 medication errors resulted in one incident report

## Slide 7
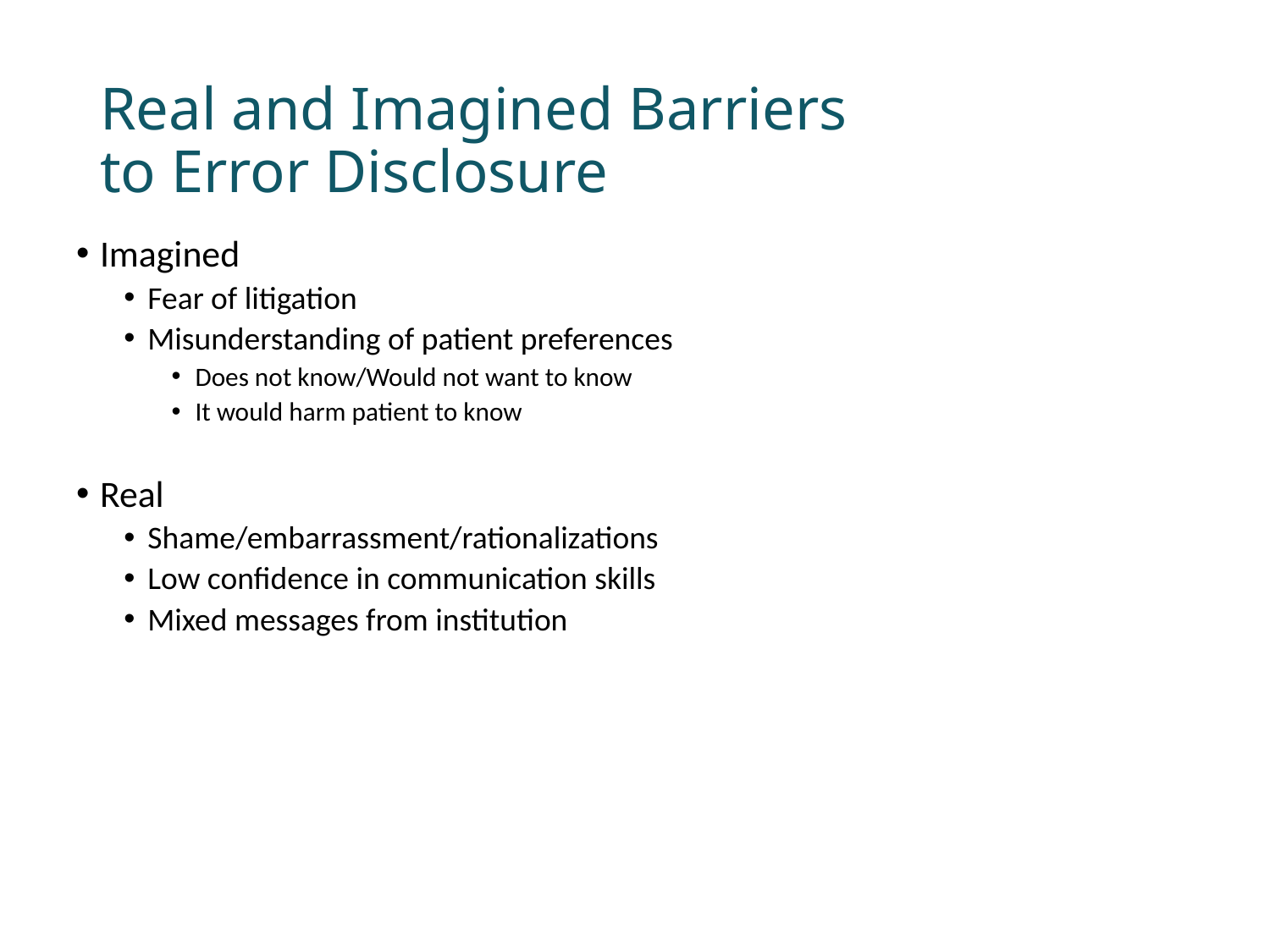

# Real and Imagined Barriers to Error Disclosure
Imagined
Fear of litigation
Misunderstanding of patient preferences
Does not know/Would not want to know
It would harm patient to know
Real
Shame/embarrassment/rationalizations
Low confidence in communication skills
Mixed messages from institution

## Slide 8
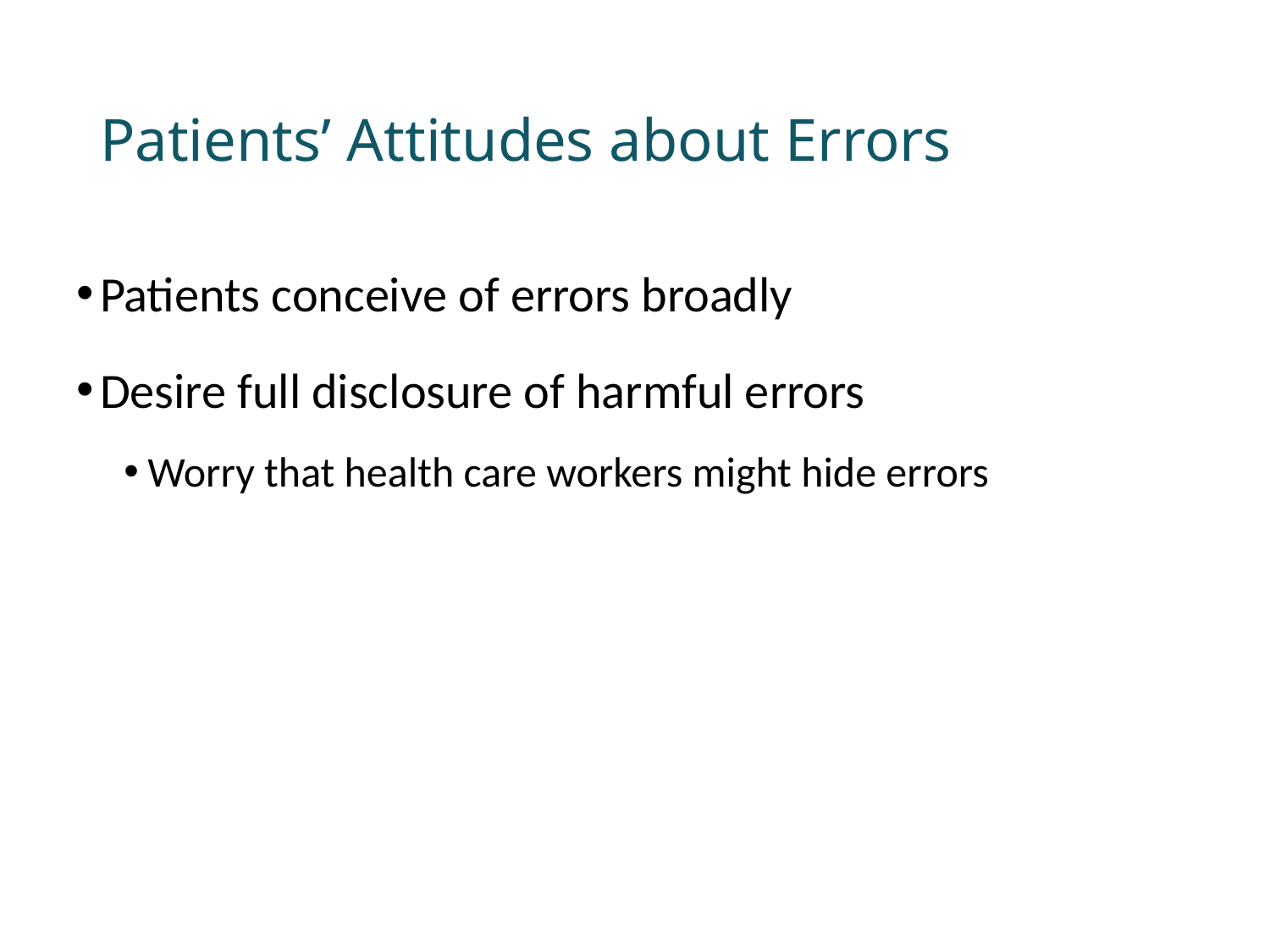

# Patients’ Attitudes about Errors
Patients conceive of errors broadly
Desire full disclosure of harmful errors
Worry that health care workers might hide errors

## Slide 9
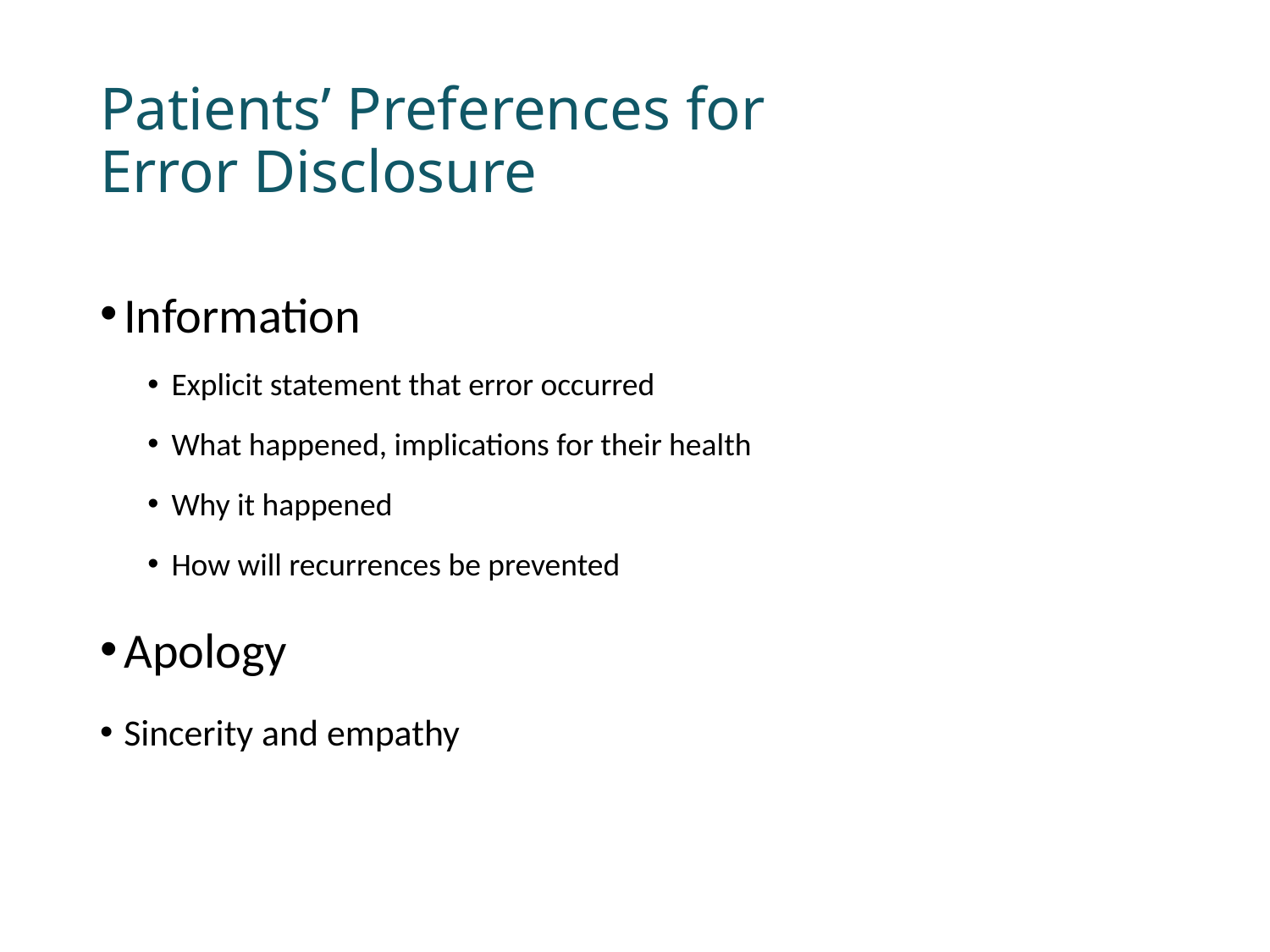

# Patients’ Preferences for Error Disclosure
Information
Explicit statement that error occurred
What happened, implications for their health
Why it happened
How will recurrences be prevented
Apology
Sincerity and empathy

## Slide 10
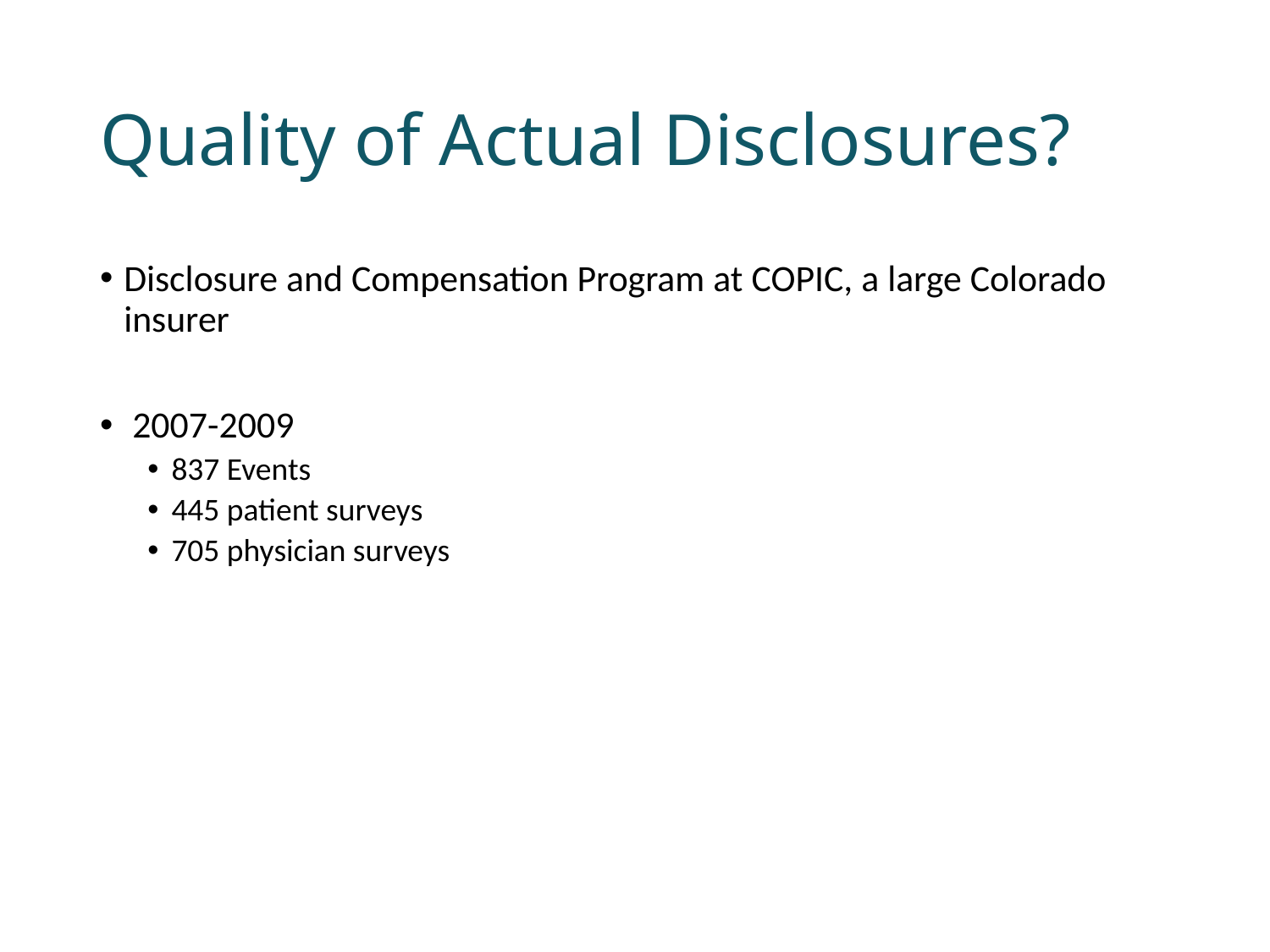

# Quality of Actual Disclosures?
Disclosure and Compensation Program at COPIC, a large Colorado insurer
 2007-2009
837 Events
445 patient surveys
705 physician surveys

## Slide 11
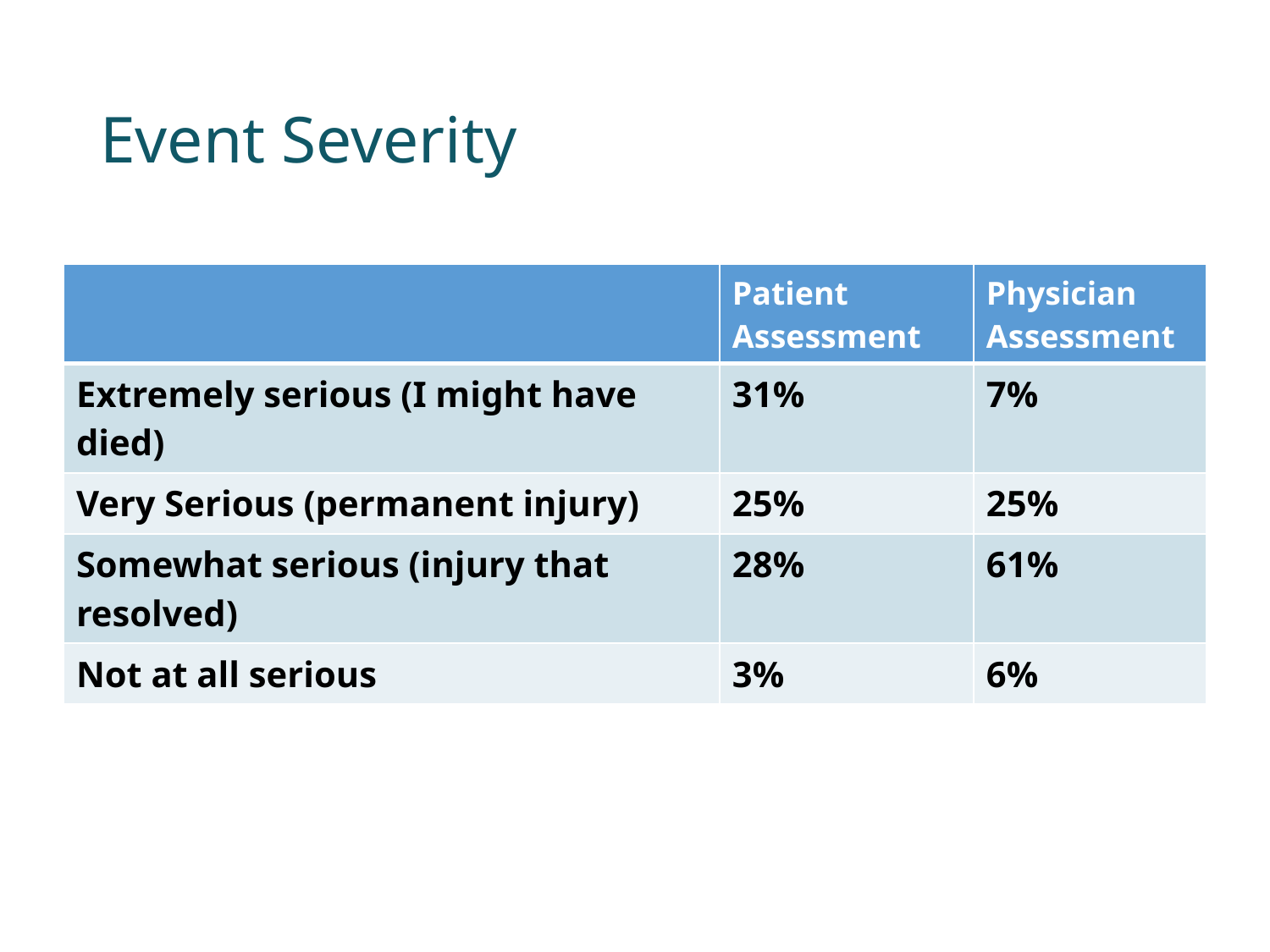

# Event Severity
| | Patient Assessment | Physician Assessment |
| --- | --- | --- |
| Extremely serious (I might have died) | 31% | 7% |
| Very Serious (permanent injury) | 25% | 25% |
| Somewhat serious (injury that resolved) | 28% | 61% |
| Not at all serious | 3% | 6% |

## Slide 12
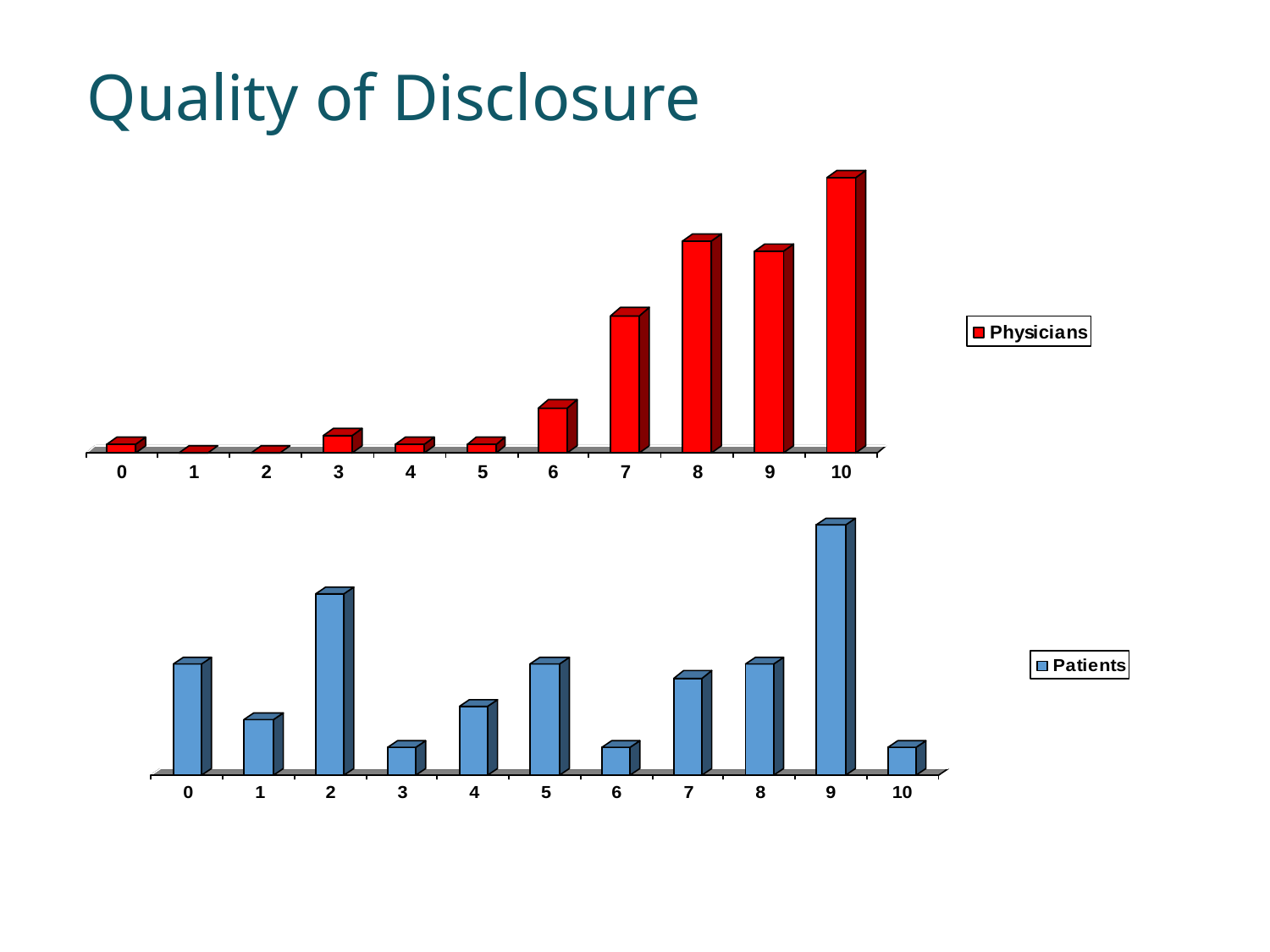

# Quality of Disclosure

## Slide 13
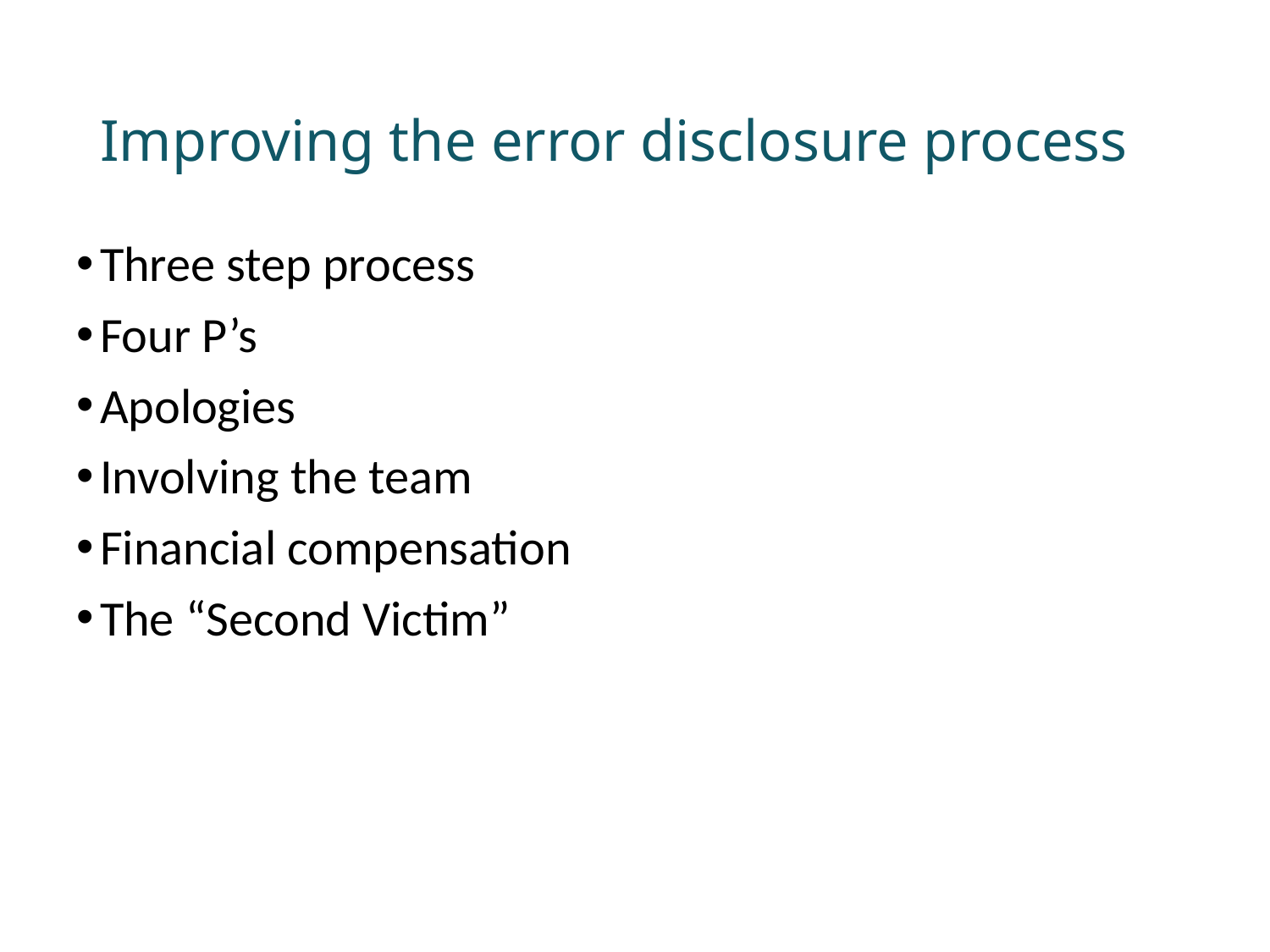

# Improving the error disclosure process
Three step process
Four P’s
Apologies
Involving the team
Financial compensation
The “Second Victim”

## Slide 14
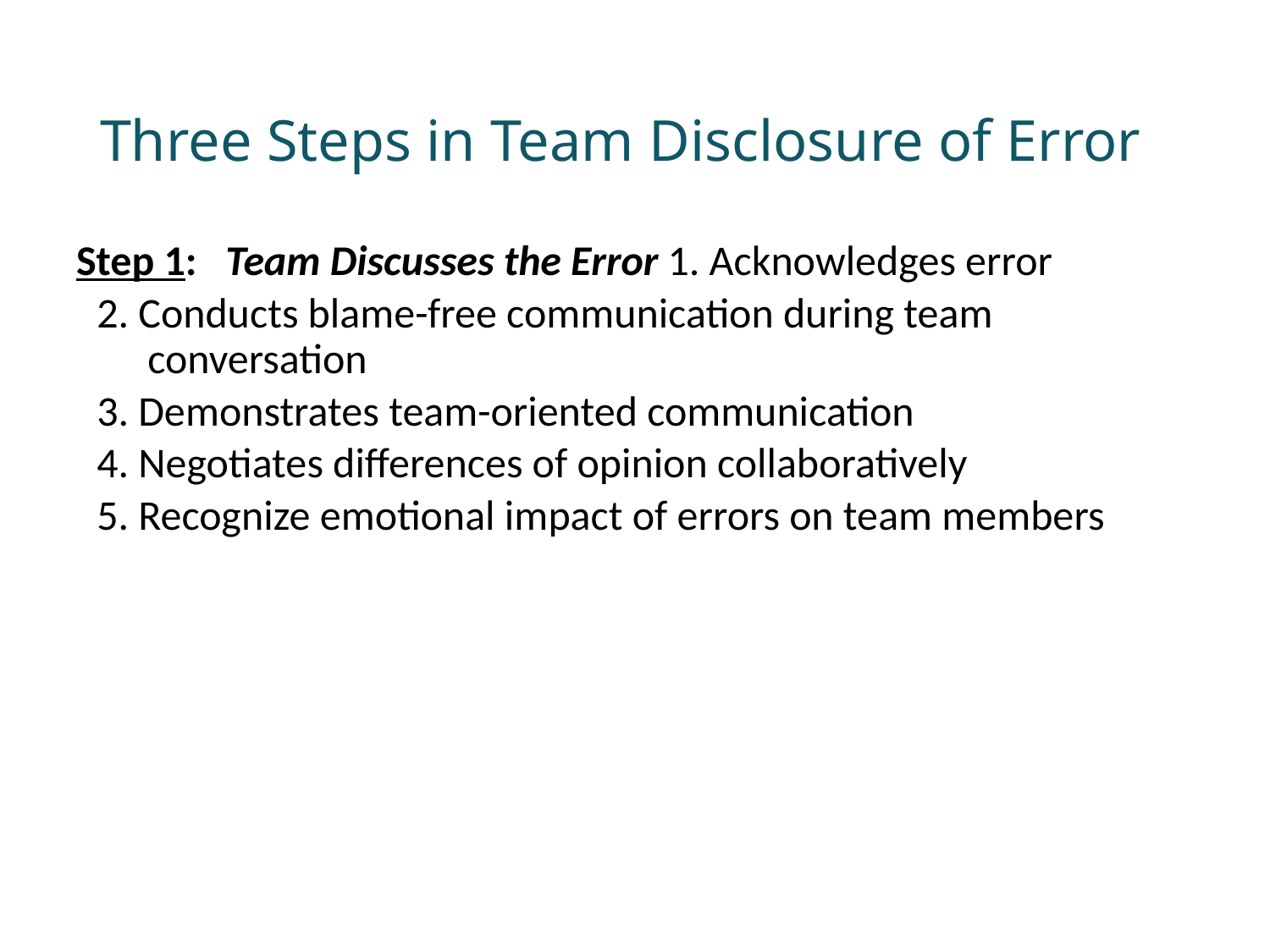

# Three Steps in Team Disclosure of Error
Step 1: Team Discusses the Error 1. Acknowledges error
2. Conducts blame-free communication during team conversation
3. Demonstrates team-oriented communication
4. Negotiates differences of opinion collaboratively
5. Recognize emotional impact of errors on team members

## Slide 15
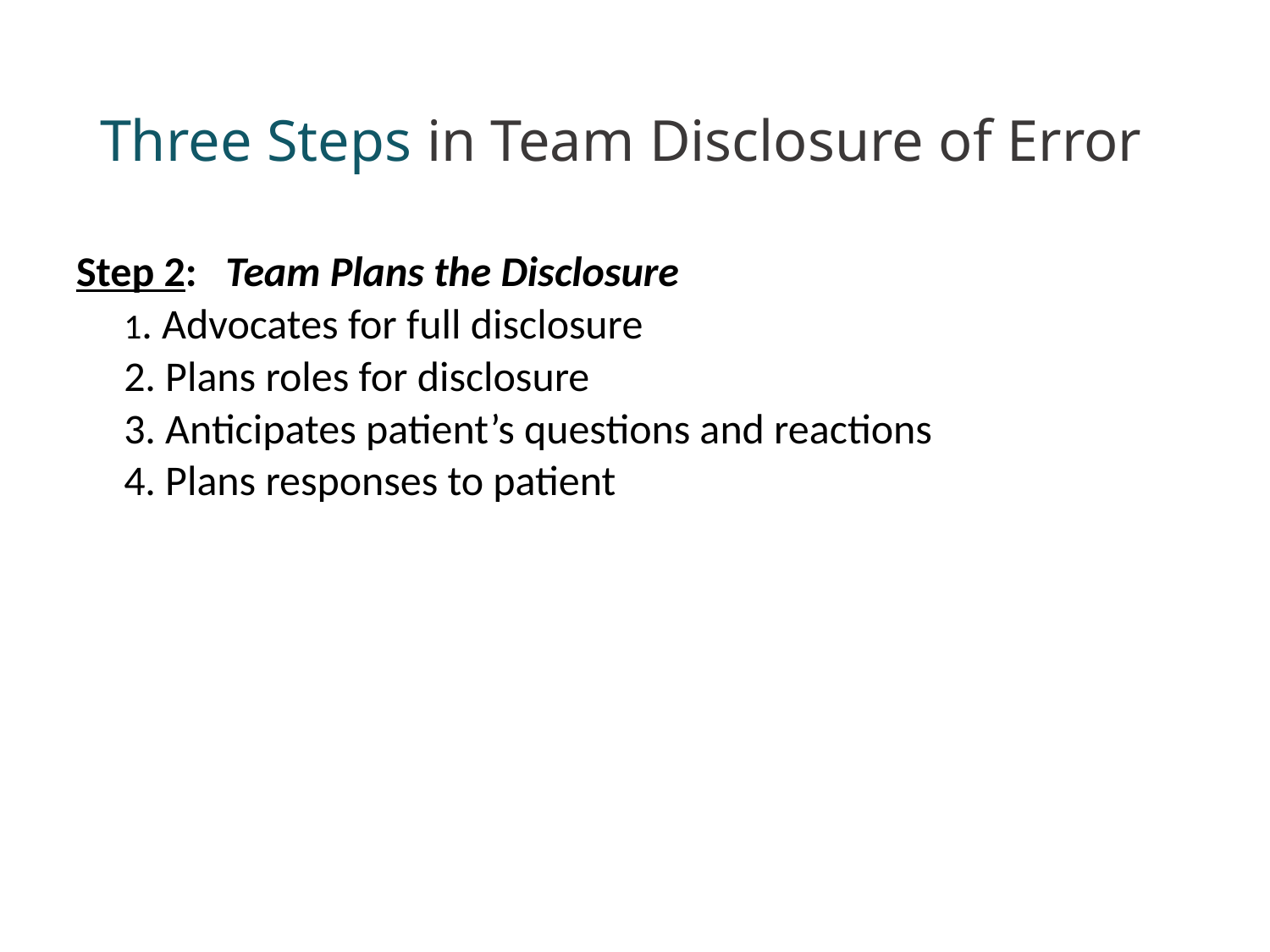

# Three Steps in Team Disclosure of Error
Step 2: Team Plans the Disclosure
1. Advocates for full disclosure
2. Plans roles for disclosure
3. Anticipates patient’s questions and reactions
4. Plans responses to patient

## Slide 16
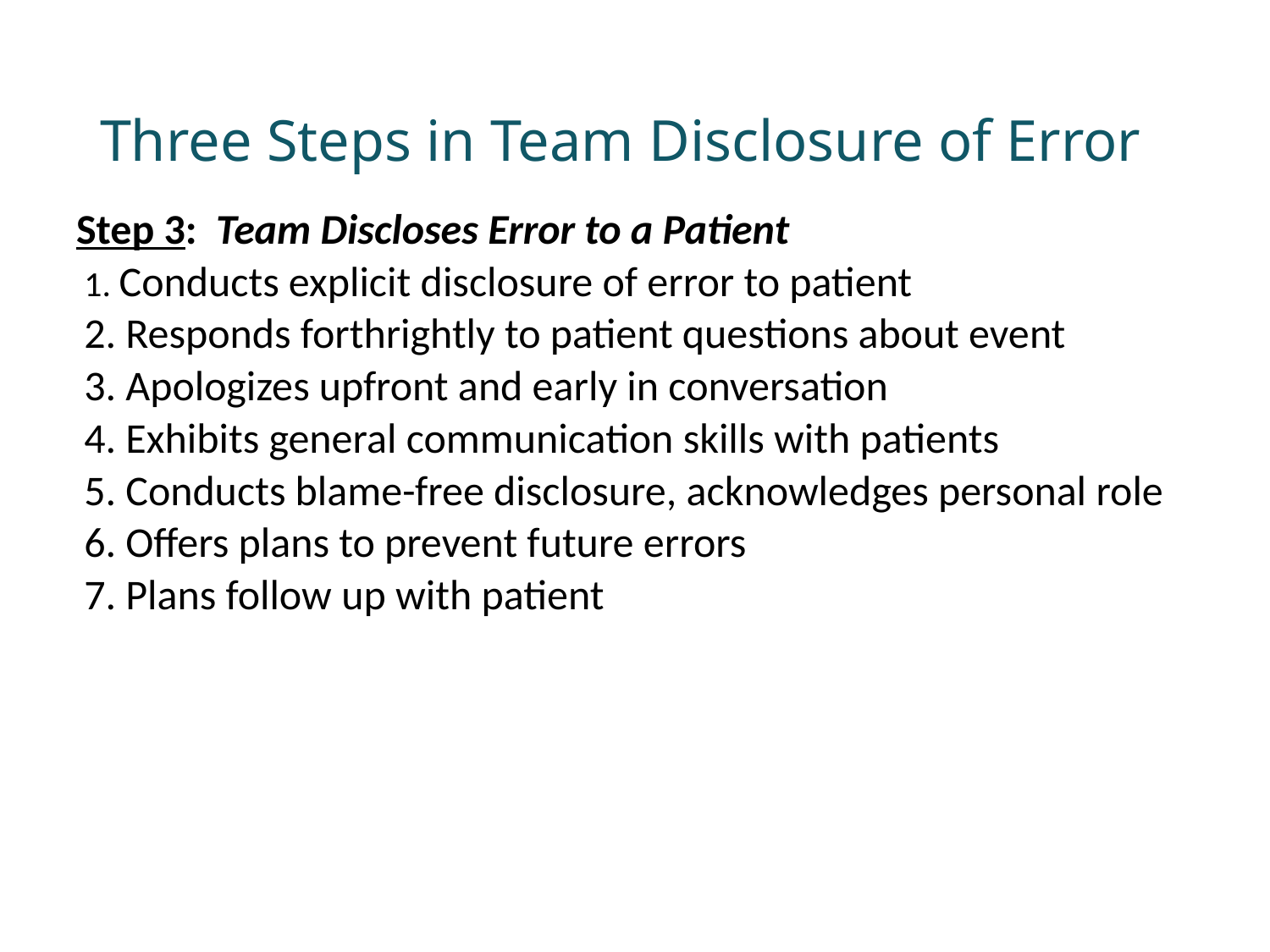

# Three Steps in Team Disclosure of Error
Step 3: Team Discloses Error to a Patient
1. Conducts explicit disclosure of error to patient
2. Responds forthrightly to patient questions about event
3. Apologizes upfront and early in conversation
4. Exhibits general communication skills with patients
5. Conducts blame-free disclosure, acknowledges personal role
6. Offers plans to prevent future errors
7. Plans follow up with patient

## Slide 17
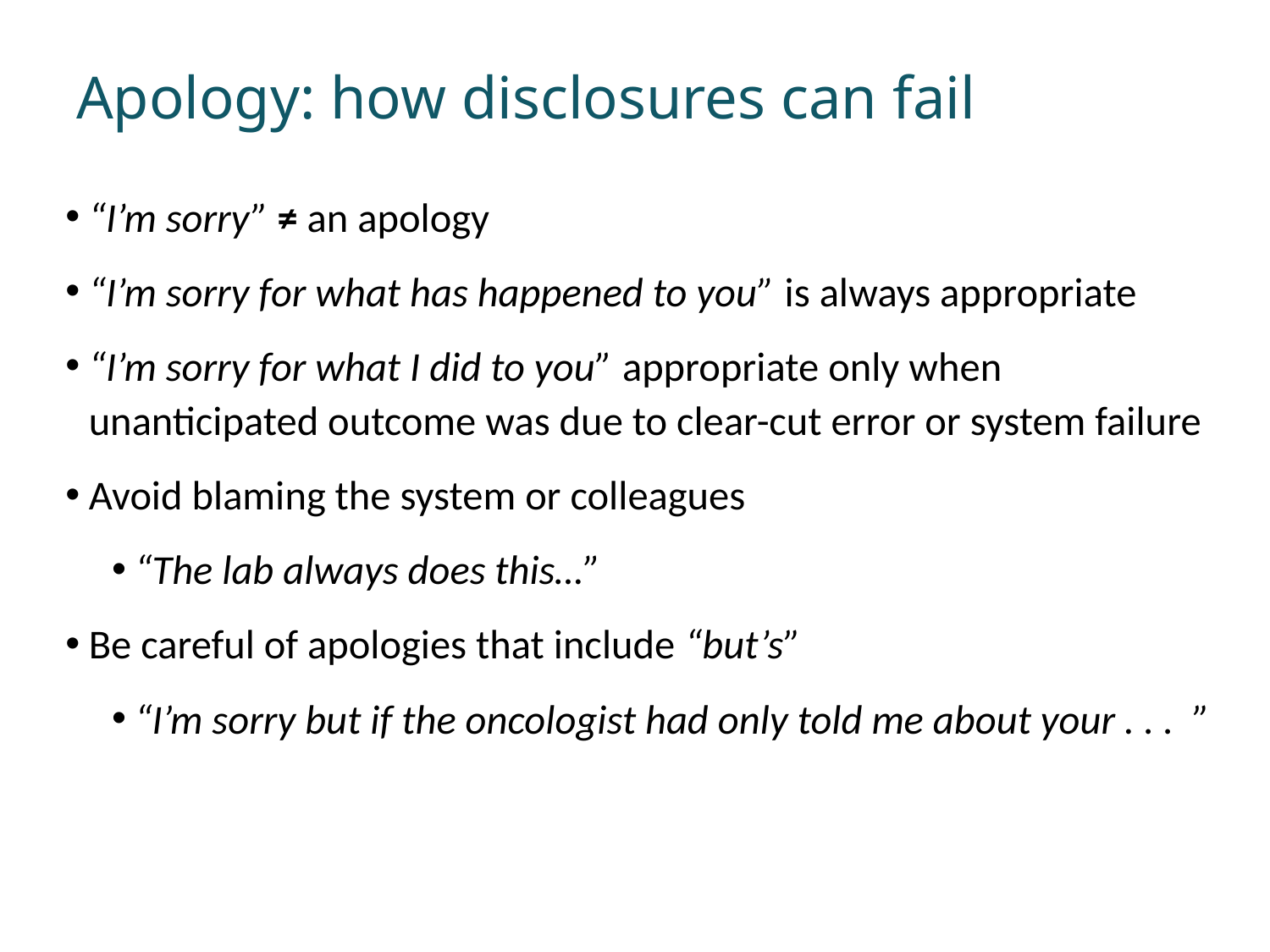

# Apology: how disclosures can fail
“I’m sorry” ≠ an apology
“I’m sorry for what has happened to you” is always appropriate
“I’m sorry for what I did to you” appropriate only when unanticipated outcome was due to clear-cut error or system failure
Avoid blaming the system or colleagues
“The lab always does this…”
Be careful of apologies that include “but’s”
“I’m sorry but if the oncologist had only told me about your . . . ”

## Slide 18
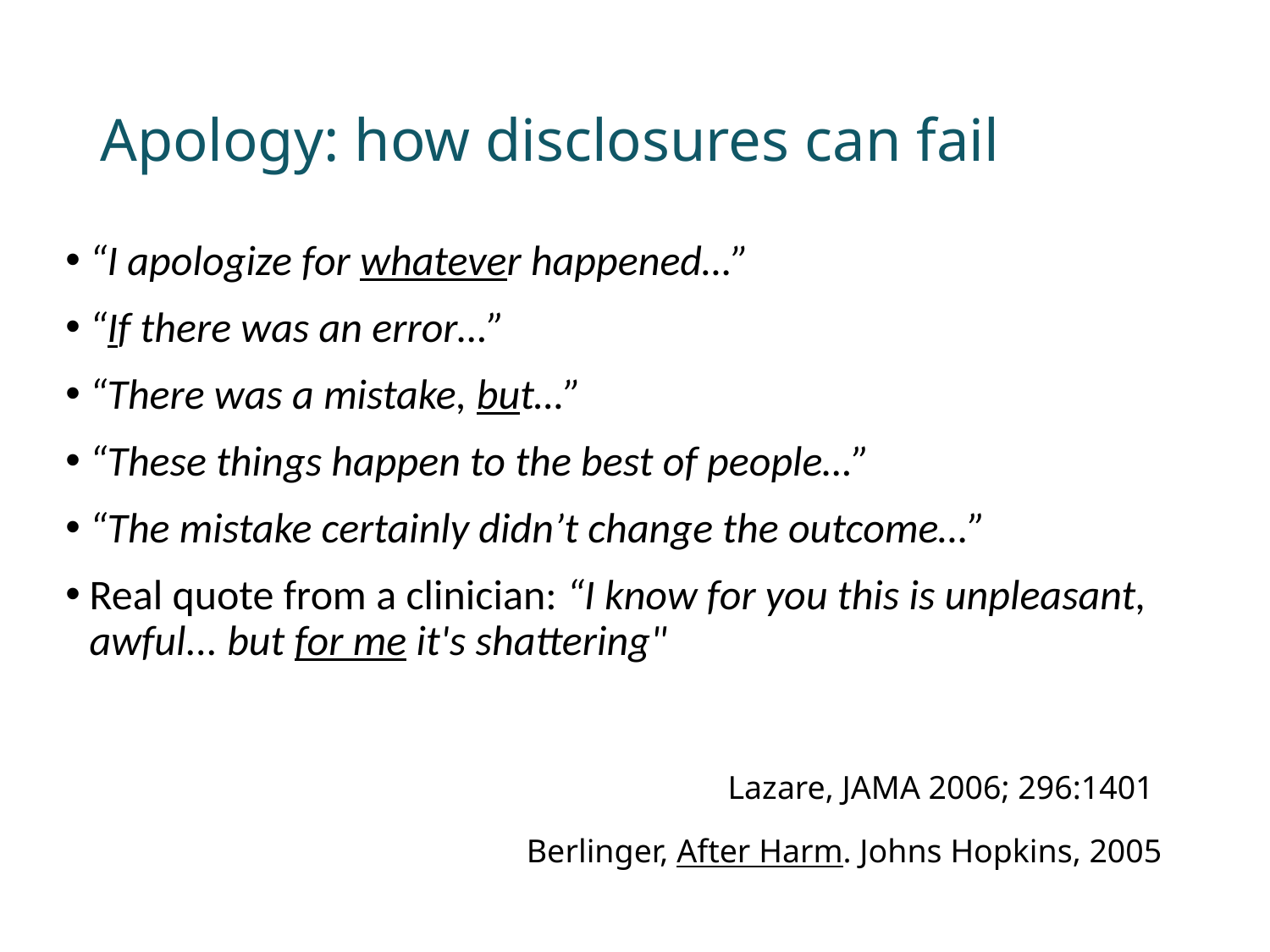

# Apology: how disclosures can fail
“I apologize for whatever happened…”
“If there was an error…”
“There was a mistake, but…”
“These things happen to the best of people…”
“The mistake certainly didn’t change the outcome…”
Real quote from a clinician: “I know for you this is unpleasant, awful... but for me it's shattering"
Lazare, JAMA 2006; 296:1401
Berlinger, After Harm. Johns Hopkins, 2005

## Slide 19
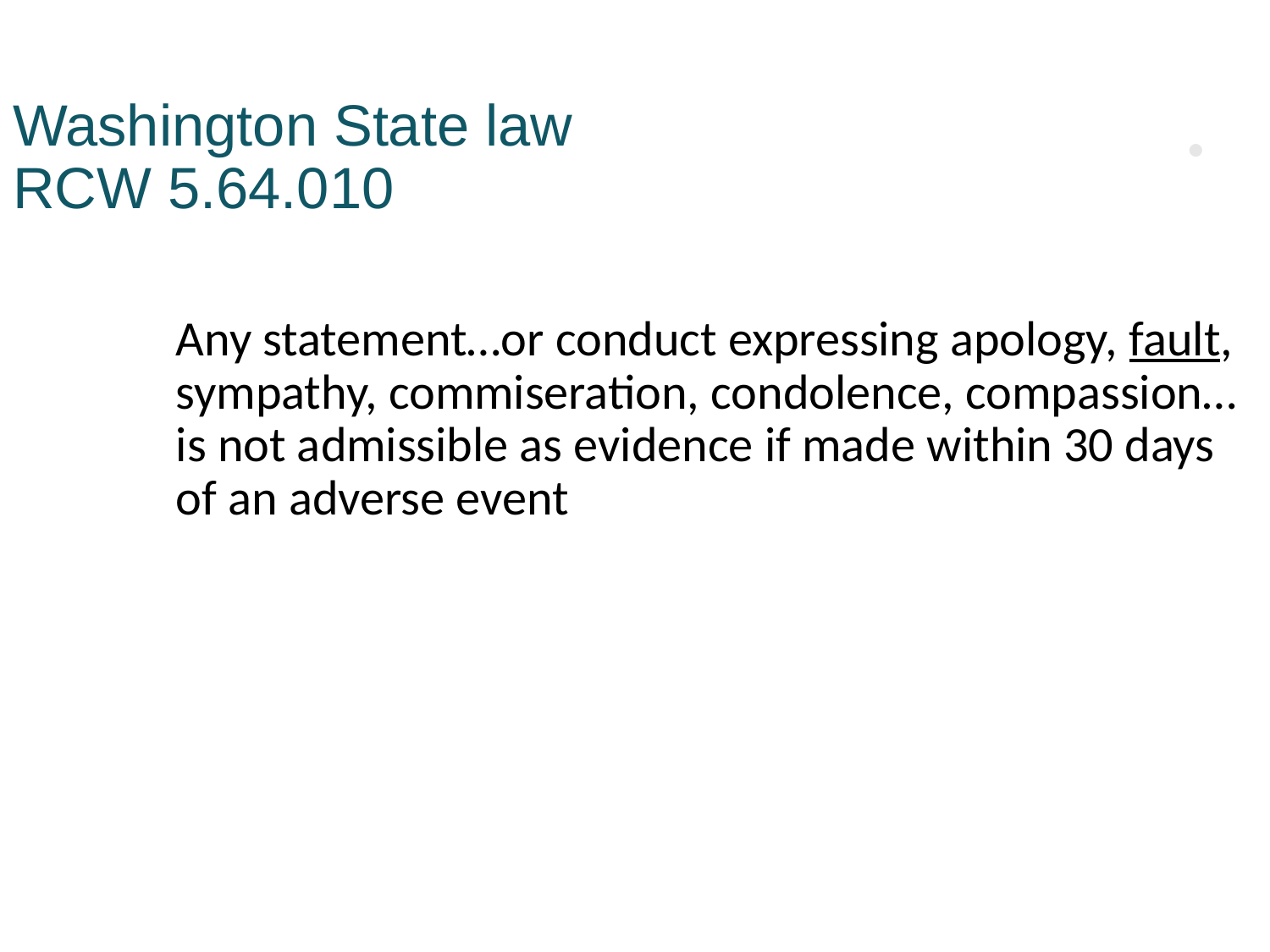

Washington State law
RCW 5.64.010
 .
Any statement…or conduct expressing apology, fault, sympathy, commiseration, condolence, compassion…is not admissible as evidence if made within 30 days of an adverse event
19

## Slide 20
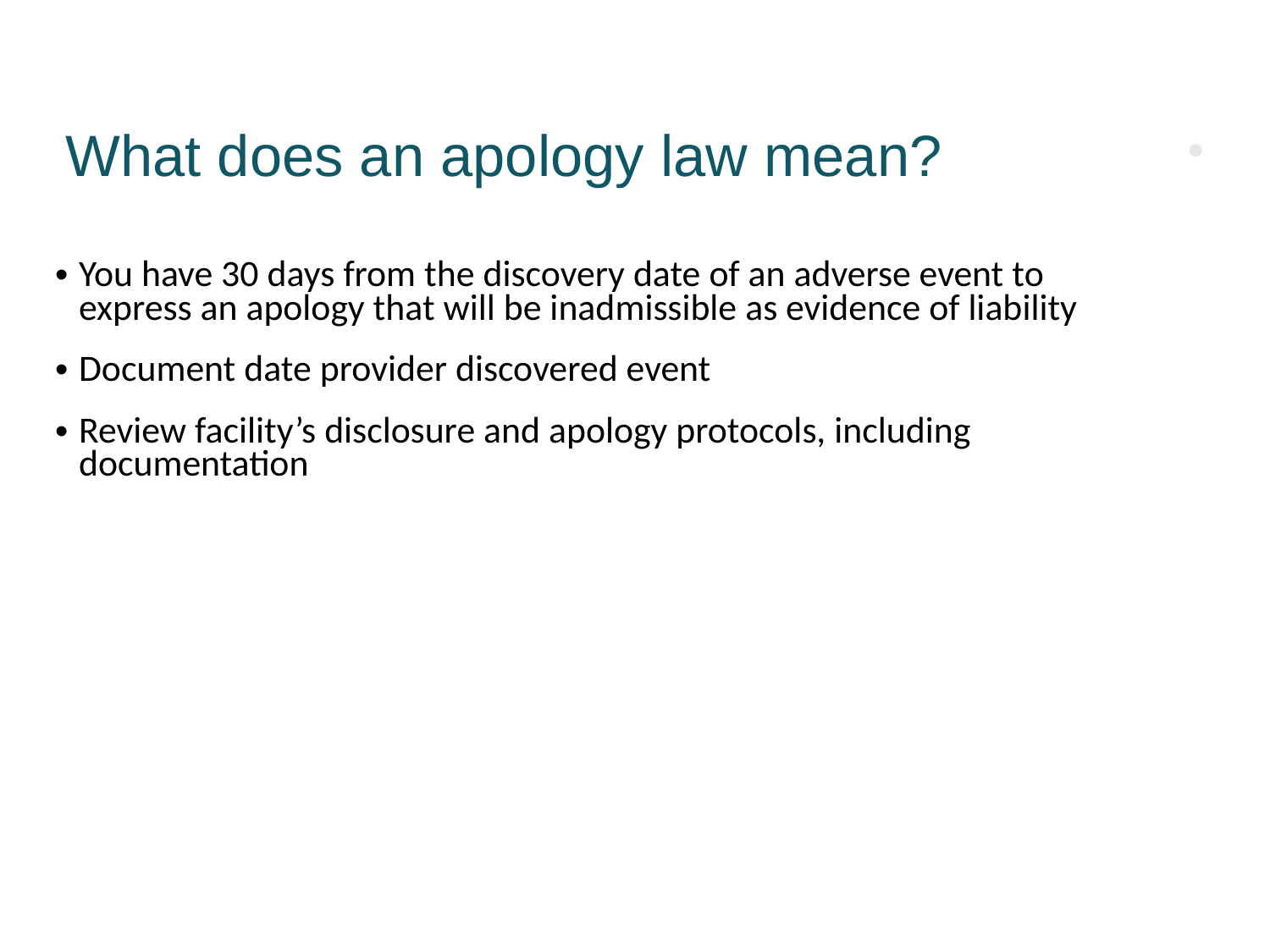

What does an apology law mean?
 .
You have 30 days from the discovery date of an adverse event to express an apology that will be inadmissible as evidence of liability
Document date provider discovered event
Review facility’s disclosure and apology protocols, including documentation

## Slide 21
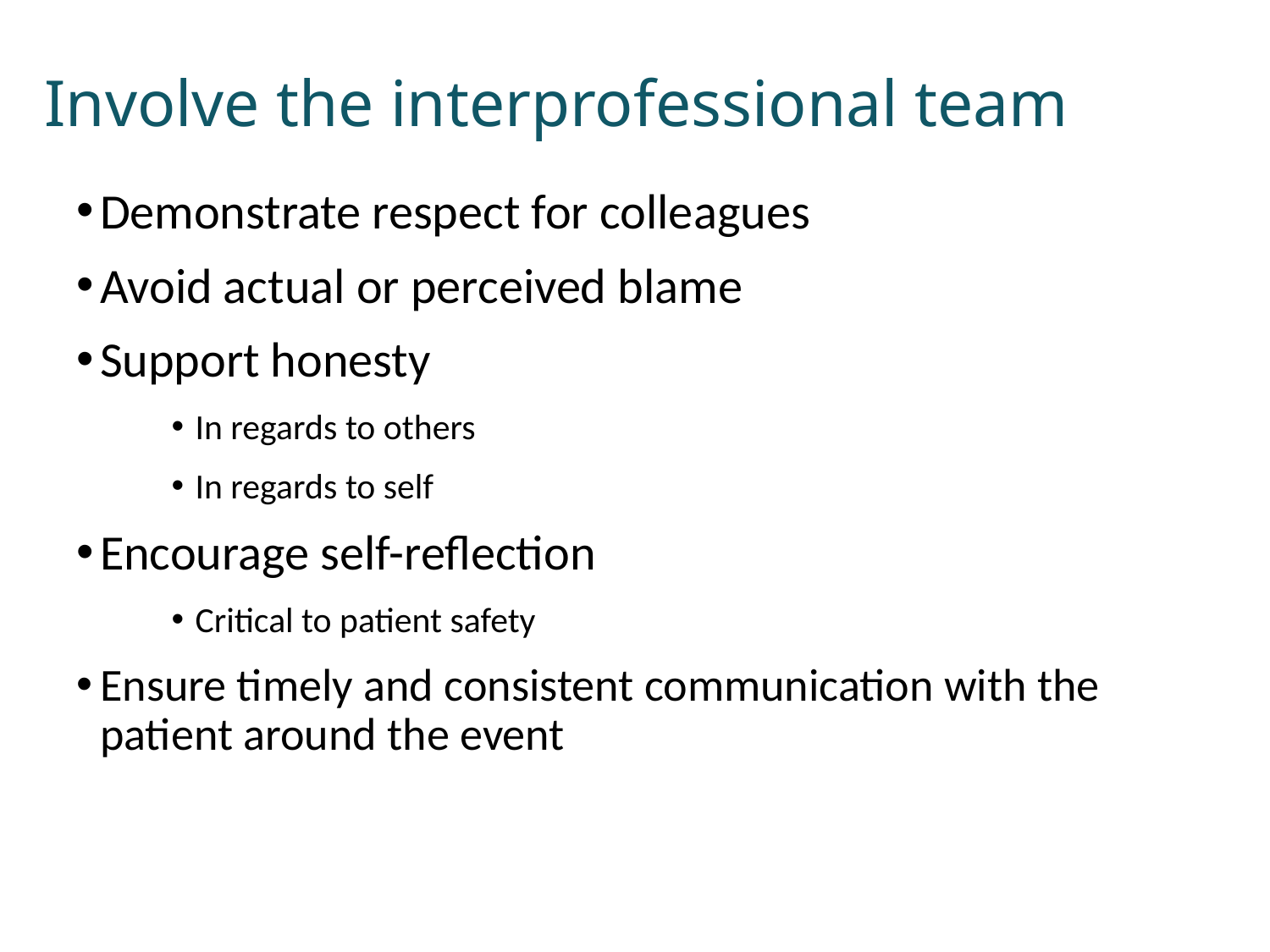

# Involve the interprofessional team
Demonstrate respect for colleagues
Avoid actual or perceived blame
Support honesty
In regards to others
In regards to self
Encourage self-reflection
Critical to patient safety
Ensure timely and consistent communication with the patient around the event

## Slide 22
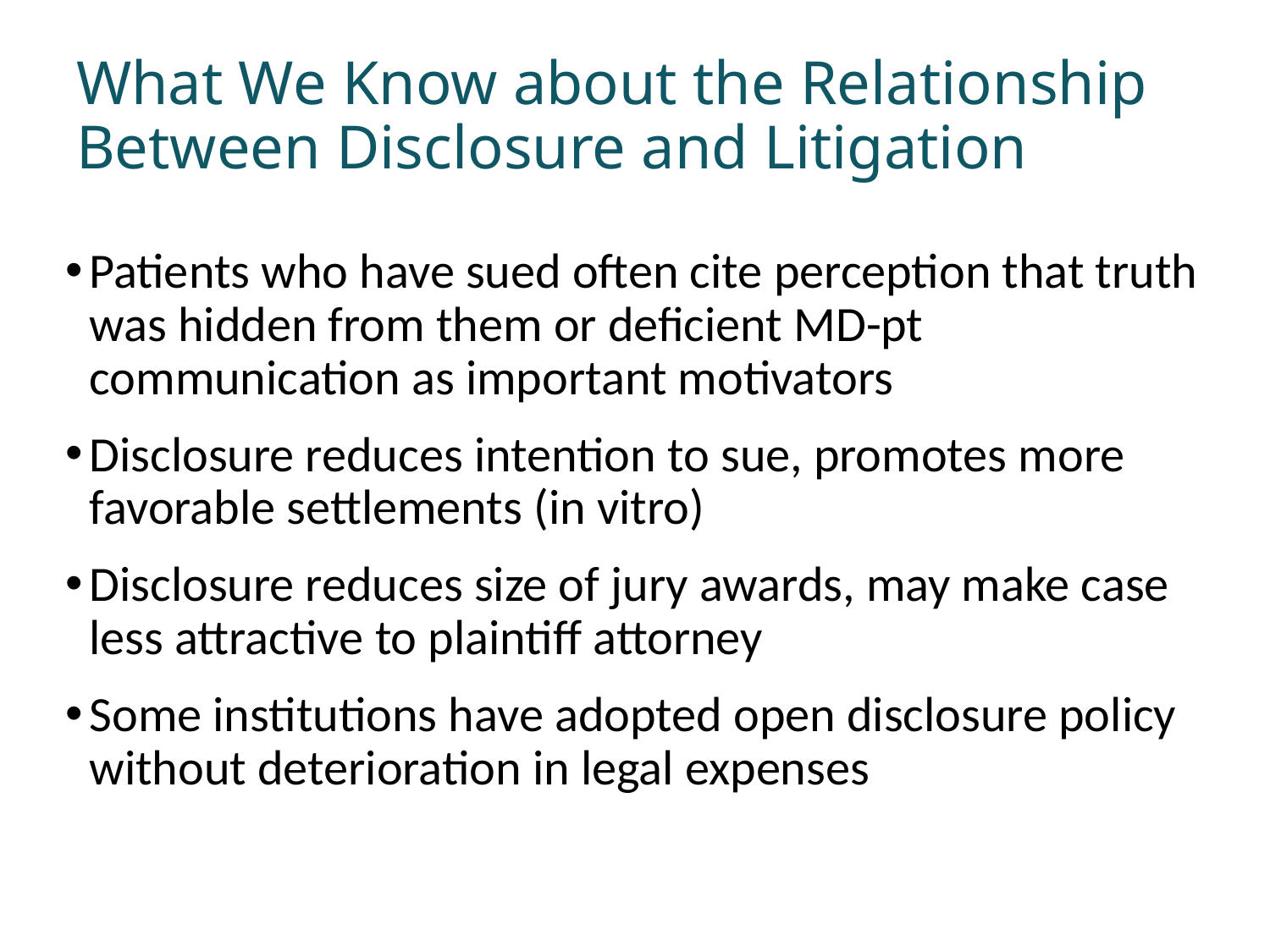

# What We Know about the Relationship Between Disclosure and Litigation
Patients who have sued often cite perception that truth was hidden from them or deficient MD-pt communication as important motivators
Disclosure reduces intention to sue, promotes more favorable settlements (in vitro)
Disclosure reduces size of jury awards, may make case less attractive to plaintiff attorney
Some institutions have adopted open disclosure policy without deterioration in legal expenses

## Slide 23
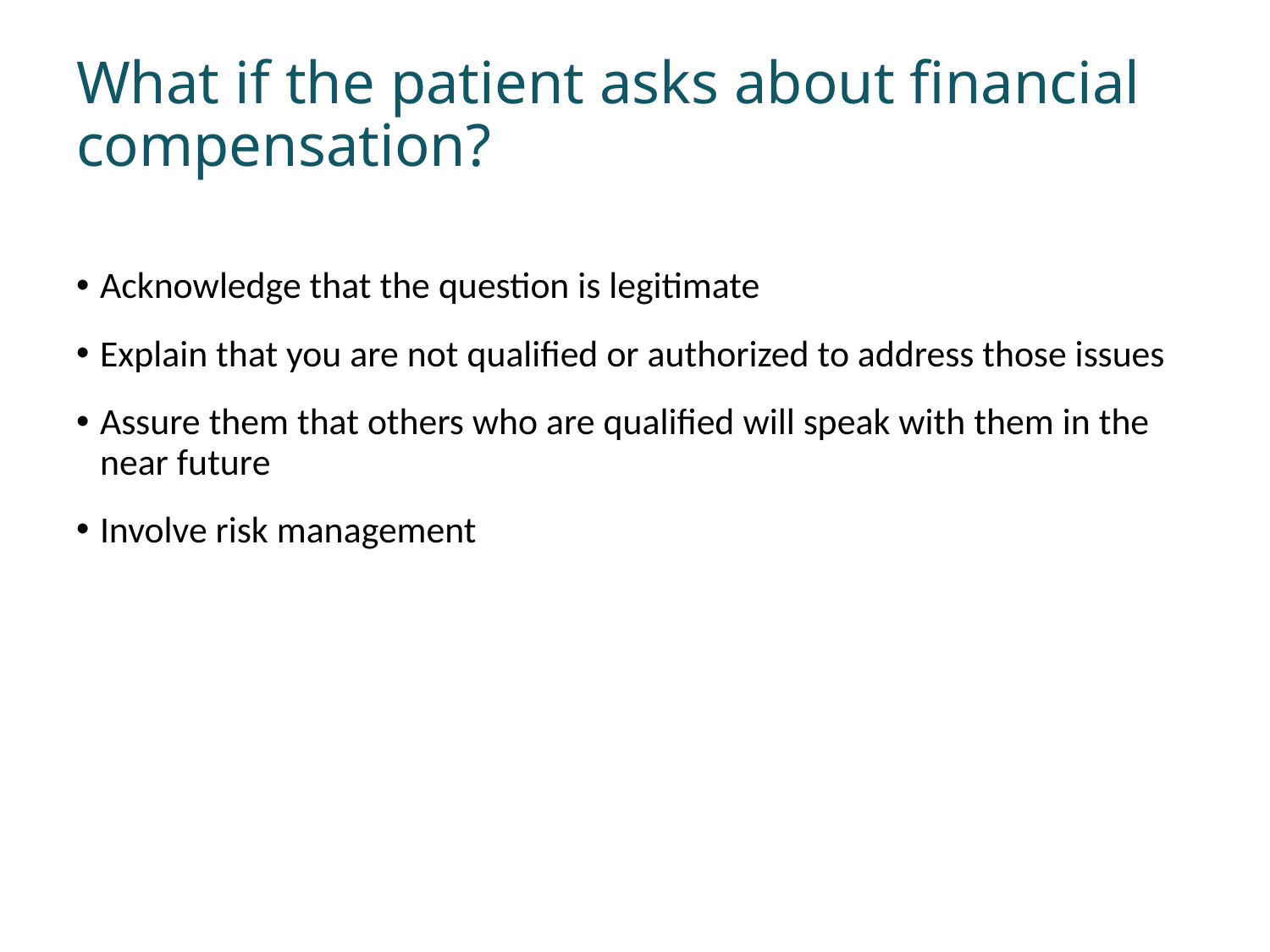

# What if the patient asks about financial compensation?
Acknowledge that the question is legitimate
Explain that you are not qualified or authorized to address those issues
Assure them that others who are qualified will speak with them in the near future
Involve risk management

## Slide 24
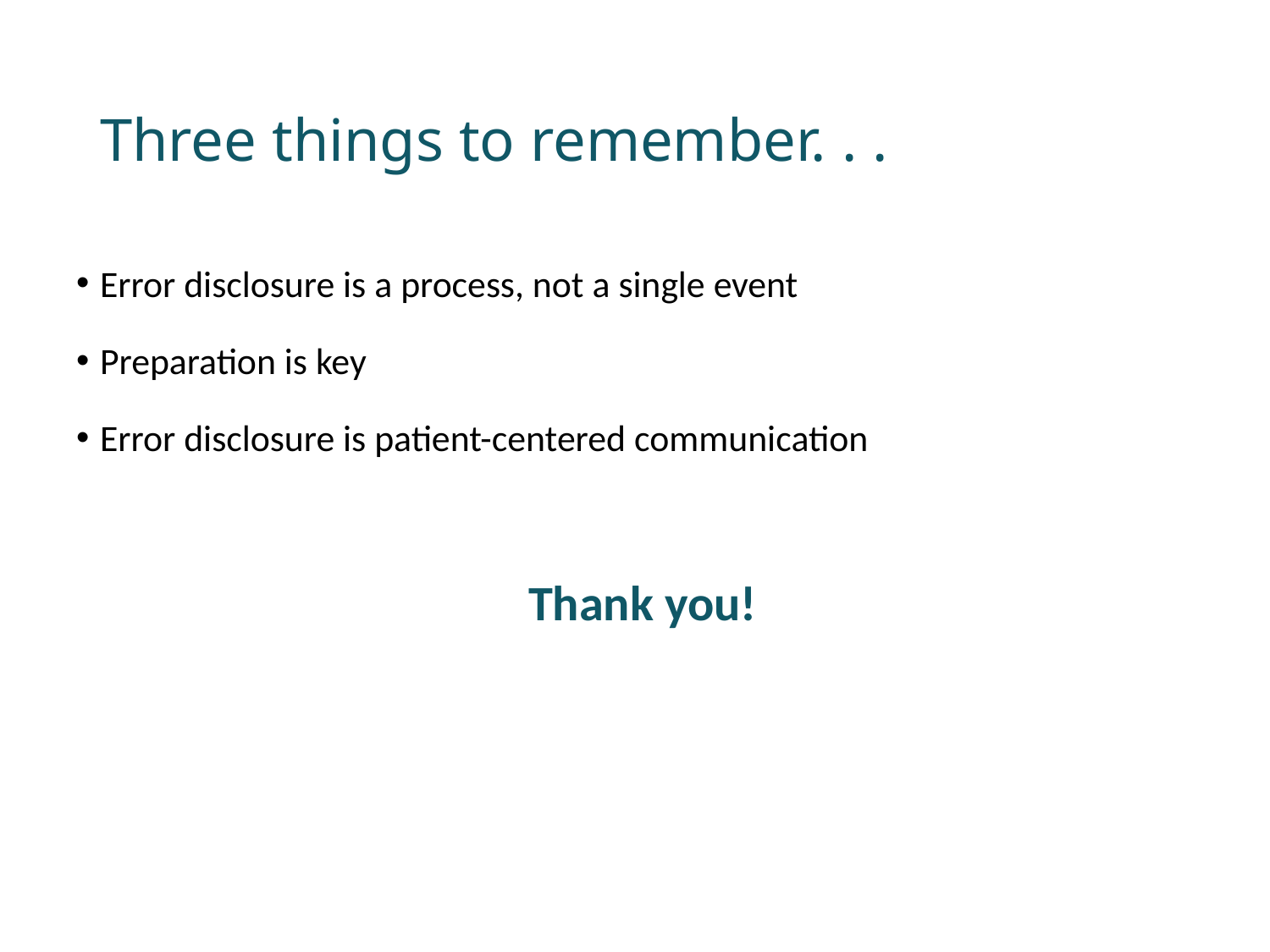

# Three things to remember. . .
Error disclosure is a process, not a single event
Preparation is key
Error disclosure is patient-centered communication
Thank you!
