# Supplementary figures and images for: Interprofessional Error Disclosure Training for Medical, Nursing, Pharmacy, Dental, and Physician Assistant Students
Source: MedEdPORTAL. 2017 Jul 21;13:10606. doi: 10.15766/mep_2374-8265.10606 (PMC6338166; doi:10.15766/mep_2374-8265.10606)

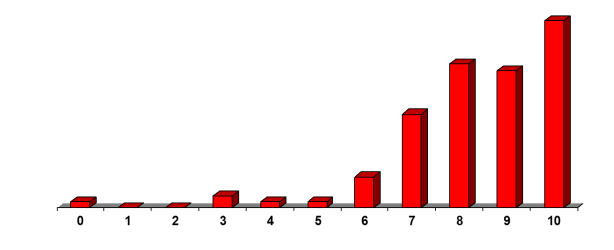

Supplement: Supplementary file 1 — A. Interprofessional Error Disclosure Module folder B. Error Disclosure Faculty Facilitators Guide.docx C. Profession-Specific Cases.docx D. Error Disclosure Pocket Cards.pdf E. Error Disclosure Slides.pptx [file mep-13-10606-s001.zip › A. Interprofessional Error Disclosure Module folder/mobile/5YUnOI01J0K_80_P_0_0_1478_583_DX1456_DY1456_CX614_CY250.png]

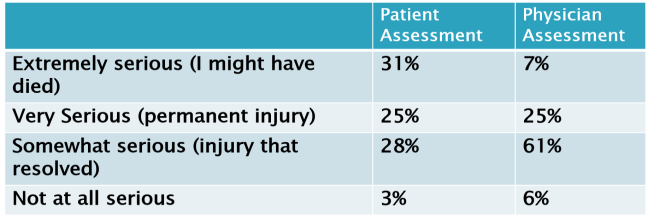

Supplement: Supplementary file 1 — A. Interprofessional Error Disclosure Module folder B. Error Disclosure Faculty Facilitators Guide.docx C. Profession-Specific Cases.docx D. Error Disclosure Pocket Cards.pdf E. Error Disclosure Slides.pptx [file mep-13-10606-s001.zip › A. Interprofessional Error Disclosure Module folder/mobile/6JNSZCaa14A_80_DX1296_DY1296_CX648_CY223.png]

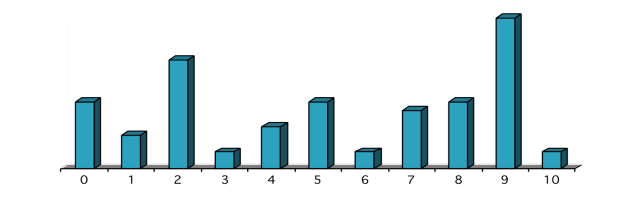

Supplement: Supplementary file 1 — A. Interprofessional Error Disclosure Module folder B. Error Disclosure Faculty Facilitators Guide.docx C. Profession-Specific Cases.docx D. Error Disclosure Pocket Cards.pdf E. Error Disclosure Slides.pptx [file mep-13-10606-s001.zip › A. Interprofessional Error Disclosure Module folder/mobile/6MRb0fdVdG6_80_P_0_0_1400_532_DX1430_DY1430_CX622_CY204.png]

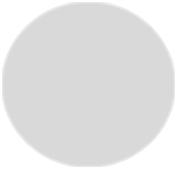

Supplement: Supplementary file 1 — A. Interprofessional Error Disclosure Module folder B. Error Disclosure Faculty Facilitators Guide.docx C. Profession-Specific Cases.docx D. Error Disclosure Pocket Cards.pdf E. Error Disclosure Slides.pptx [file mep-13-10606-s001.zip › A. Interprofessional Error Disclosure Module folder/mobile/txt__default_5ipA6tuLHJj.png]

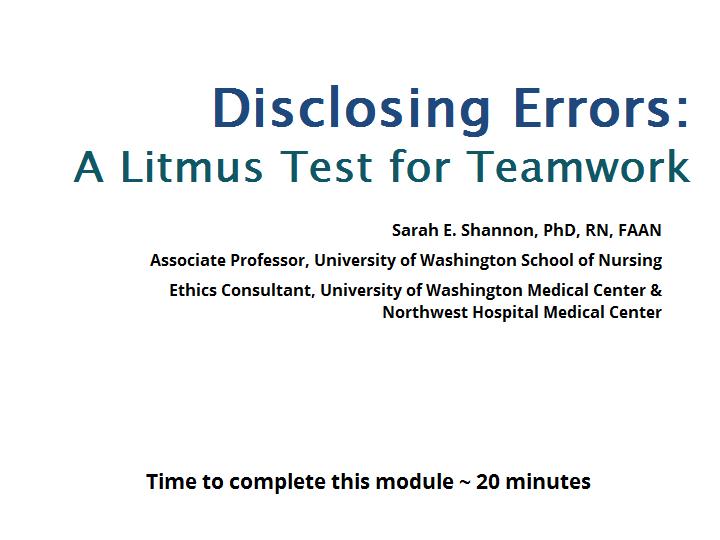

Supplement: Supplementary file 1 — A. Interprofessional Error Disclosure Module folder B. Error Disclosure Faculty Facilitators Guide.docx C. Profession-Specific Cases.docx D. Error Disclosure Pocket Cards.pdf E. Error Disclosure Slides.pptx [file mep-13-10606-s001.zip › A. Interprofessional Error Disclosure Module folder/story_content/thumbnail.jpg]
